# Description of a new catfish genus (Siluriformes, Loricariidae) from the Tocantins River basin in central Brazil, with comments on the historical zoogeography of the new taxon

**DOI:** 10.3897/zookeys.598.7400

**Published:** 2016-06-14

**Authors:** Gabriel S. C. Silva, Fábio F. Roxo, Luz E. Ochoa, Claudio Oliveira

**Affiliations:** 1 Laboratório de Biologia e Genética de Peixes, Departamento de Morfologia, IBB–UNESP, Campus de Botucatu, 18618–970, Botucatu, São Paulo state, Brazil

**Keywords:** Molecular phylogeny, Freshwater fishes, headwater capture, catfish, taxonomy

## Abstract

This study presents the description of a new genus of the catfish subfamily Neoplecostominae from the Tocantins River basin. It can be distinguished from other neoplecostomine genera by the presence of (1) three hypertrophied bicuspid odontodes on the lateral portion of the body (character apparently present in mature males); (2) a large area without odontodes around the snout; (3) a post-dorsal ridge on the caudal peduncle; (4) a straight tooth series in the dentary and premaxillary rows; (5) the absence of abdominal plates; (6) a conspicuous series of enlarged papillae just posterior to the dentary teeth; and (7) caudal peduncle ellipsoid in cross section. We used maximum likelihood and Bayesian methods to estimate a time-calibrated tree with the published data on 116 loricariid species using one nuclear and three mitochondrial genes, and we used parametric biogeographic analyses (DEC and DECj models) to estimate ancestral geographic ranges and to infer the colonization routes of the new genus and the other neoplecostomines in the Tocantins River and the hydrographic systems of southeastern Brazil. Our phylogenetic results indicate that the new genus and species is a sister taxon of all the other members of the Neoplecostominae, originating during the Eocene at 47.5 Mya (32.7–64.5 Mya 95% HPD). The present distribution of the new genus and other neoplecostomines may be the result of a historical connection between the drainage basins of the Paraguay and Paraná rivers and the Amazon basin, mainly through headwater captures.

## Introduction

The Loricariidae, an endemic Neotropical family of freshwater fish, is the largest group of catfish, with about 900 valid species (Eschmeyer and Fong 2015). Within the Loricariidae, the subfamily Neoplecostominae has a long complex taxonomic and systematic history, with a number of major morphological and molecular studies being conducted since the nineteenth century (e.g. Eigenmann and Eigenmann 1890; Regan 1904; Gosline 1947; Isbrücker 1980; Howes 1983; Schaefer 1987; Montoya-Burgos et al. 1998; Armbruster 2004; Chiachio et al. 2008; Roxo et al. 2012a, 2014).

The neoplecostomines are small-bodied catfishes which were, until now, restricted to southern and southeastern Brazil, where they are found in small- to medium-sized streams with clear and shallow water, of up to 1 m in depth (Langeani 1990). Previous studies (e.g. Chiachio et al. 2008; Roxo et al. 2012a, 2014) concluded that the considerable diversity of this subfamily can be accounted for primarily by the geomorphological processes (i.e. tectonics and erosion) that have shaped the South American continent over the past 100 Mya, influencing fish distribution and speciation patterns (Ribeiro 2006; Albert and Reis 2011). In this context, one of the principal processes is river capture (also known as stream capture or headwater capture), an important landscape-level mechanism that can isolate lineages and promote diversification (Waters et al. 2006; Winemiller et al. 2008; Albert and Crampton 2010) by changing the connectivity of adjacent river basins (Smith 1981; Hocutt and Wiley 1986; Mayden 1988; Lundberg et al. 1998). The consequences of this process for the local fauna can be profound, changing watershed boundaries and allowing previously isolated species to disperse and colonize new environments (Grant et al. 2007; Muneepeerakul et al. 2008; Bertuzzo et al. 2009).

Here, we recognize a new genus and species of neoplecostomine catfish based on specimens collected during a recent expedition to the Tocantins River basin in Goiás state, Brazil. The new taxon is described in detail below.

## Material and methods

### Morphological analysis

Body plate nomenclature follows Schaefer (1997) and measurements, Armbruster (2003), except for the dorsal-adipose distance, adipose-spine length, dorsal adipose-caudal distance, ventral adipose-caudal distance, adipose-anal distance and mouth width. Measurements and counts were taken on the left side of the specimens and were taken point to point, to the nearest 0.1 mm with digital calipers. Specimens were cleared and stained (c&s) according to the method of Taylor and Van Dyke (1985). Dorsal fin ray counts include the spinelet as the first unbranched ray. Counts of vertebrae include the five vertebrae that comprise the Weberian apparatus, while the compound caudal centrum (PU1 + U1) was counted as a single element. Zoological nomenclature follows the International Code of Zoological Nomenclature (International Commission on Zoological Nomenclature 1999).

### Molecular analysis

#### Taxon sampling

The molecular analysis included 157 specimens representing 116 loricariid species (115 species from the study of Roxo et al. [2014], and one sample of the new genus, see Suppl. material 1 for all taxa). *Diplomystes mesembrinus* (Ringuelet, 1982) was used as the outgroup to root all phylogenies (Arratia 1987; de Pinna 1993, 1998; Grande 1987; Grande and de Pinna 1998; Mo 1991; Sullivan et al. 2006). Samples of *Corydoras imitator* Nijssen & Isbrücker, 1983, *Corydoras oiapoquensis* Nijssen, 1972, *Hoplosternum littorale* (Hancock, 1828), *Callichthys callichthys* (Linnaeus, 1758), *Astroblepus* spp. 1 and 2, *Hemipsilichthys gobio* (Lütken, 1874), *Hemipsilichthys papillatus* Pereira, Oliveira & Oyakawa, 2000, *Delturus parahybae* Eigenmann & Eigenmann, 1889b, *Rineloricaria lanceolata* (Günther, 1868), *Spatuloricaria* sp. 1, *Hypostomus ancistroides* (Ihering, 1911), *Hypostomus nigromaculatus* (Schubart 1964) and *Hypostomus microstomus* Weber, 1987 were also included in the analysis as outgroups.

Vouchers of the samples were those catalogued by Roxo et al. (2014), except for the samples of the new genus, which was deposited in the collection of Auburn University Natural History Museum
(AUM), Auburn; Laboratório de Biologia e Genética de Peixes (LBP), Botucatu; and Museu de Zoologia da Universidade de São Paulo
(MZUSP), São Paulo.

#### DNA extraction and sequencing

Total DNA was extracted from muscle samples collected from two specimens of the new genus preserved in ethanol using the protocol described by Aljanabi and Martinez (1997). Partial sequences for two genes, Cytochrome B (CytB), forward 5’-CCA TCC AAC ATC TCA GCA TGA TGA AA 3’, reverse 5’-AAC CTC CGA TCT TCG GAT TAC AAG AC 3` (Oliveira et al. 2011), and 16S rRNA, forward 5’-ACG CCT GTT TAT CAA AAA CAT-3’, reverse 5’-CCG GTC TGA ACT CAG ATC ACG T-3’ (Kocher et al. 1989) were amplified by polymerase chain reaction (PCR). The amplification was conducted in a total volume of 12.5 µl with 1.25 µl of 10 X buffer (10 mM Tris-HCl+15 mM MgCl2), 0.5 µl of the dNTPs (200 nM of each), 0.5 µl of each 5 mM primer, 0.05 µl of platinum Taq polymerase (Invitrogen), 1 µl of template DNA (12 ng), and 8.7 µl of dd H_2_O. The PCR reactions consisted of 30–40 cycles, 30 s at 95 °C, 15–30 s at 48–58 °C, and 45–90 s at 72 °C. All the PCR products were first identified visually on a 1% agarose gel and then purified using ExoSap-IT (USB Corporation) following the manufacturer’s instructions. The purified PCR products were sequenced using the Big DyeTM Terminator v 3.1 Cycle Sequencing Ready Reaction kit (Applied Biosystems), purified by ethanol precipitation and loaded into a 3130-Genetic Analyzer automatic sequencer (Applied Biosystems).

#### Sequencing and phylogenetic analysis

The individual sequences of each species were initially analyzed in the BioEdit 5.0.9 software (Hall 1999), and a consensus sequence was obtained for each DNA segment. The sequences were then aligned in MUSCLE (Edgar 2004) using the default parameters, and inspected visually. To evaluate the saturation of the matrix by substitution, we calculated the index of substitution saturation (Iss), as described by Xia et al. (2003) and Xia and Lemey (2009), and the transition/transversion rate, in DAMBE 5.2.31 (Xia and Xie 2001). The Iss was calculated without taking gaps into account.

Maximum likelihood analyses were run in RAxML Web-Servers (Stamatakis et al. 2008). Bootstrap (BS) resampling (Felsenstein 1985) was used to evaluate the support for each node, based on 1000 replicates. Random starting trees were used for each independent ML tree search, while all other parameters were set at the default values. The ML analyses were based on the GTR model.

### Time calibration and estimates of ancestral ranges

The uncorrelated relaxed (lognormal) molecular clock was calibrated using BEAST v.1.7.5. All clade-age estimates are presented as the mean and 95% highest posterior density (HPD) values. We included two calibration points to constrain the divergence dates for the 157 clades identified in our phylogenetic tree. The first calibration point was implemented as a normally-distributed prior, with an offset of 125 million years ago (Mya), and a standard deviation of 15 million years. Data from the stratigraphic record and the geographic distribution of living taxa indicate that the Siluriformes originated during the Lower Cretaceous (145–100 Mya; Lundberg 1993; Sullivan et al. 2006; Lundberg et al. 2007).

The second calibration point was implemented using a log-normal prior set at 55 Mya, with a mean and standard deviation of 1 for the origin of the family Callichthyidae. The oldest known callichthyid fossil, *Corydoras revelatus* Cockerell (1925) was dated to the Paleocene by Marshall et al. (1997), assuming 55 Mya as a minimum age. We used a macroevolutionary Birth–Death model to estimate diversification likelihood values, with a starting tree obtained from the RAxML analysis. These analyses were conducted under the GTR model. The ML tree obtained in this analysis was used as a starting tree for the MCMC searches. This analysis was run for 100 million generations and sampled every 10,000th generation. Stationarity and the sufficient mixing of parameters (ESS>200) were verified using Tracer v1.5 (Rambaut and Drummond 2007a). A consensus tree was built in TreeAnnotator v1.7.5 (Rambaut and Drummond 2007b).

Data on the geographic distribution of the species in each of the three subfamilies analyzed here (Hypoptopomatinae, Neoplecostominae and Otothyrinae) were obtained from the original species descriptions and the catalog of Eschmeyer (2015), with the species classification following Roxo et al. (2014). Species ranges are located within five biogeographic regions: A, Drainage basins of the Atlantic coast of southeastern Brazil; B, Upper Paraná basin; C, Paraguay, Lower Paraná and Uruguay basins; D, Amazon and Orinoco basins; E, São Francisco basin and the coastal drainage basins of northeastern Brazil.

We estimated the likelihood of ancestral range evolution using the Dispersal-Extinction-Cladogenesis (DEC: Ree and Smith 2008) and jumping (DECj: Matzke 2013a) models of species range evolution. These models are composed of two (DEC) or three (DECj) parameters including: 1) dispersal (D), where ancestral ranges expand by adding new geographic units, 2) extinction (E), where ancestral ranges are reduced by extirpating geographic units, and 3) jumping events (j), where j specifies the weight of the jumping events beyond an ancestral range (Matzke 2014). The two models of range evolution (i.e. DEC and DECj) were implemented in the R package BioGeoBEARS (Matzke 2013b). The global likelihood of the six biogeographic scenarios found using the two models (i.e. DEC and DEC+J models) were compared using the Akaike Information Criterion (AIC) (Akaike 1973) (Suppl. material 2). The model that obtained the lowest AIC values was model 2 with the DEC+J model (M2 – DEC + J), which constrained the dispersal rates between adjacent areas at 1.0 and areas separated by one or more intercalated areas at 0.5.

## Results

### Microplecostomus gen. n.

Taxon classificationAnimaliaSiluriformesLoricariidae

http://zoobank.org/077BD513-6BF2-47D9-AB4C-4A496FE33115

Figs 1 , 6

#### Type species.

*Microplecostomus forestii* sp. n.

#### Diagnosis.

**Figure 1.**
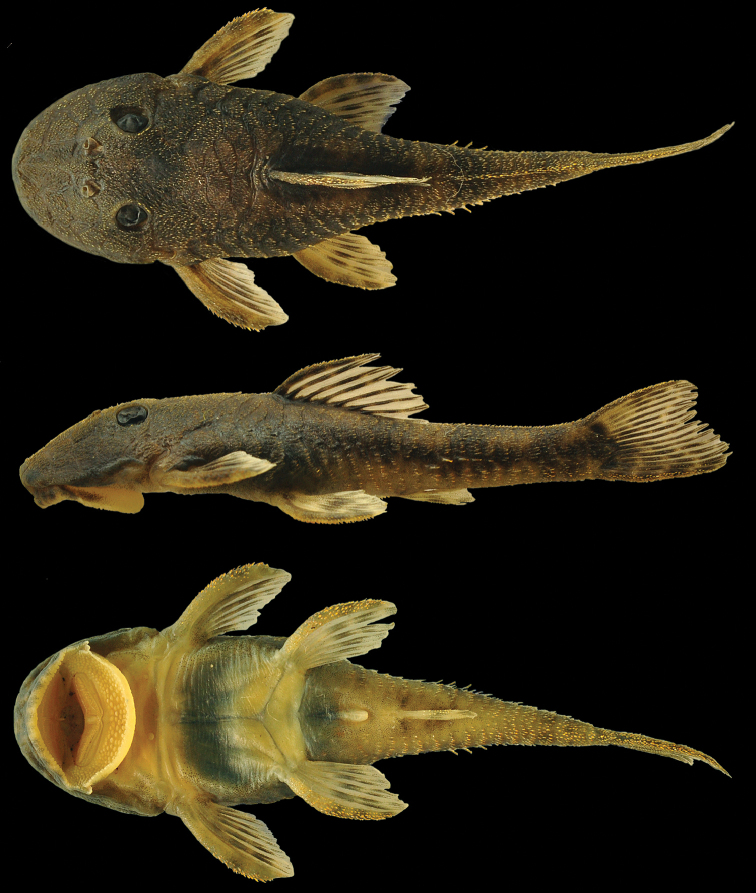
*Microplecostomus forestii* sp. n., MZUSP 118673, holotype, male, 38.3 mm SL, Goiás state, Brazil, Tocantins River basin.

**Figure 2.**
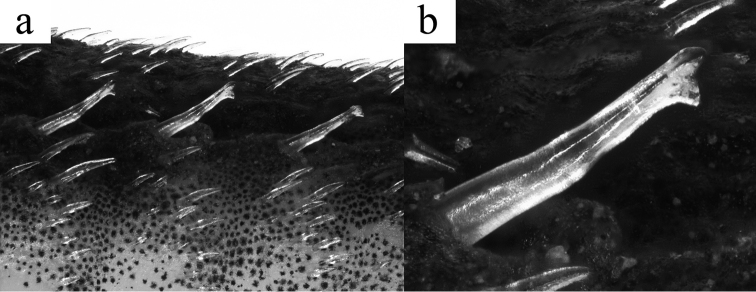
Photographs showing **a** the three hypertrophied bicuspid odontodes on the lateral portion of the body of the holotype of *Microplecostomus forestii* sp. n., MZUSP 118673; **b** Detail of the hypertrophied bicuspid odontodes.

**Figure 3.**
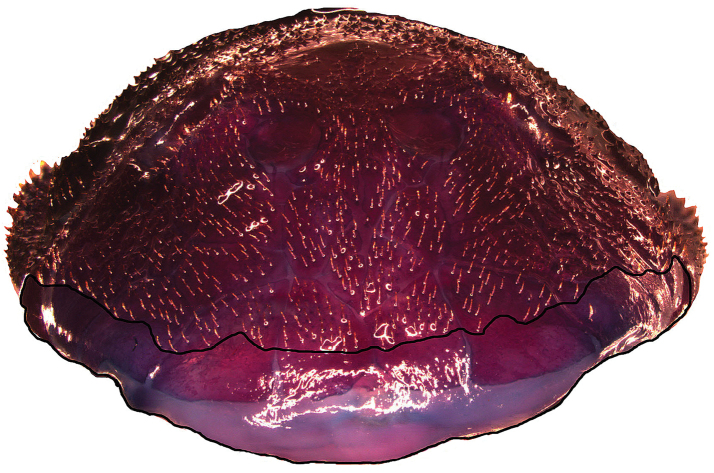
*Microplecostomus forestii* sp. n. showing a large area without odontodes around the snout, LBP 19017, 29.0 mm SL.

**Figure 4.**
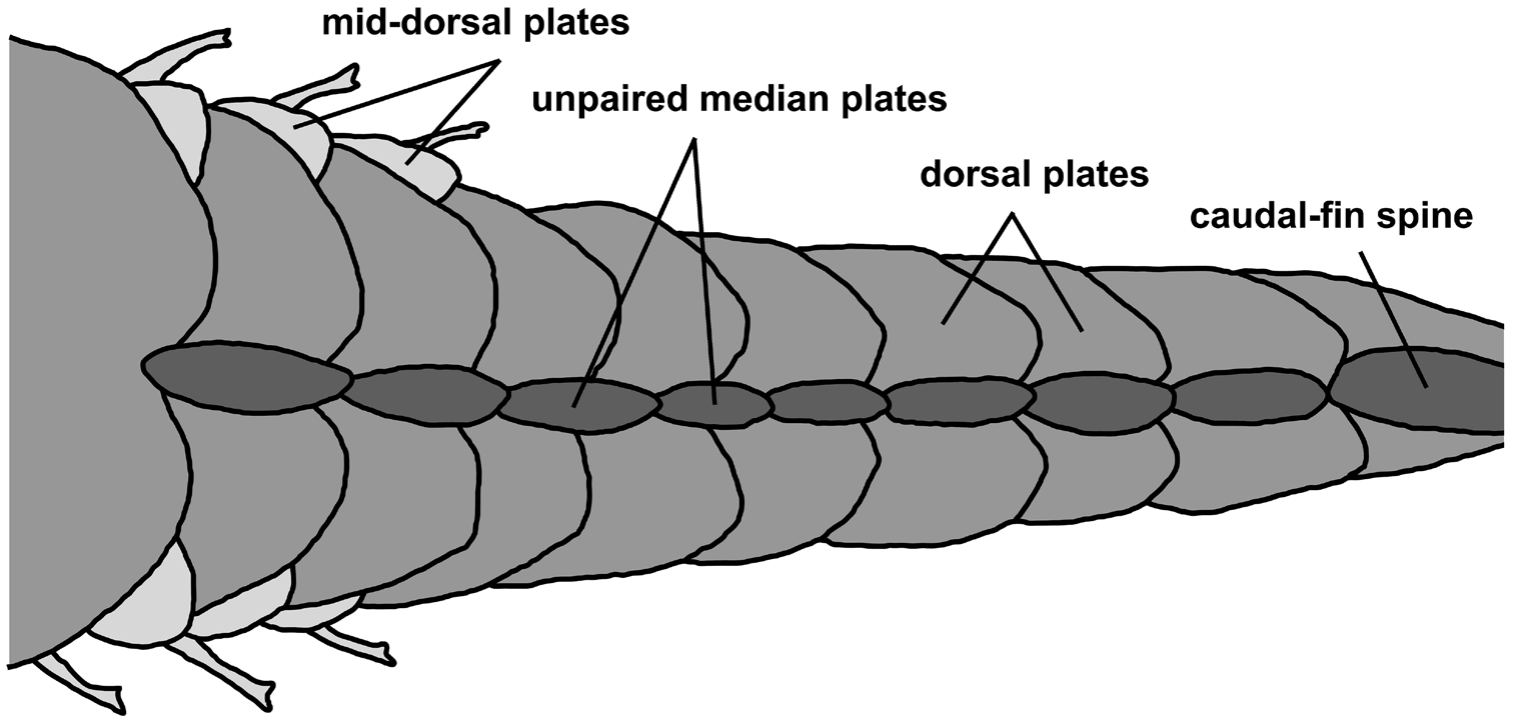
Dorsal view of the caudal peduncle in *Microplecostomus forestii* sp. n., LBP 19017, 29.0 mm SL, showing the presence of a series of unpaired median plates that form a post-dorsal ridge.

The new genus and species differs from all members of the Loricariidae by having (1) three hypertrophied bicuspid odontodes on the lateral portion of the body (character apparently present only in mature males – observed in the holotype, but not present in the paratypes) (Fig. 2a, b); and differs from all members of the Neoplecostominae by having (2) a large area without odontodes around the snout, observed in all specimens, Fig. 3 (*vs.* margin of snout bearing odontodes); and from all members of the Neoplecostominae, except *Hirtella carinata* Pereira, Zanata, Cetra & Reis, 2014, *Pareiorhina carrancas* Bockmann & Ribeiro, 2003 and *Pareiorhina hyptiorhachis* Silva, Roxo & Oliveira, 2013 by (3) the presence of a post-dorsal ridge on the caudal peduncle, see dorsal view of holotype in Figs 1, 4 (*vs.* the absence of a post-dorsal ridge). *Microplecostomus forestii* sp. n. differs from species of the genera *Isbrueckerichthys*, *Neoplecostomus* and *Pseudotocinclus* by (4) the absence of abdominal plates, Fig. 1 (*vs.* abdomen covered by pentagonal or hexagonal platelets); from *Kronichthys* by having (5) the tooth series in dentary and premaxillary rows straight (*vs.* tooth series strongly curved medially); from *Neoplecostomus* by (6) the absence of a conspicuous series of enlarged papillae just posterior to the dentary teeth (*vs.* presence of enlarged papillae); and from *Pseudotocinclus* by having (7) the caudal peduncle ellipsoid in cross section (*vs.* caudal peduncle square in cross-section).

#### Etymology.

The generic name is a combination of Greek, *micro* (mikrós) = small, related to the small size of the adult type-specimen, and *plecostomus* = a former generic name of species currently included in Loricariidae, also in reference to the small adult size of the type-species. A masculine name.

### Microplecostomus forestii sp. n.

Taxon classificationAnimaliaSiluriformesLoricariidae

http://zoobank.org/2A1A0D93-ED90-4C5F-9562-D3209D951630

Figs 1 , 6 ; Table 1

#### Holotype.

MZUSP 118673 (adult male, 38.3 mm SL), Brazil, Goiás state, municipality of São João D’Aliança, Roncador Stream, a tributary of das Brancas Stream, tributary of the Tocantizinho River, Tocantins River basin, 14°53'47.2"S, 47°34'58.4"W, 9 November 2014, FF Roxo, GSC Silva, LEO Ochoa, LH Roxo.

#### Paratypes.

All from Brazil, Goiás state, Tocantins River basin (15 specimens). AUM 67015, 1, 29.4 mm SL, municipality of Água Fria de Goiás, córrego das Brancas, tributary of rio Tocantizinho, 14°53'47.2"S, 47°34'58.4"W, 9 November 2014, FF Roxo, GSC Silva, LEO Ochoa, LH Roxo. LBP 17318, 2, 24.2–30.3 mm SL, municipality of São João D’Aliança, Roncador Stream, a tributary of das Brancas Stream, 14°43'51.3"S, 47°32'34.0"W, 21 November 2012, BF Melo, GSC Silva, JHM Martinez, R Devidé. LBP 19000, 2, 29.8–32.2 mm SL, collected with the holotype. LBP 19017, 1, 24.8 mm SL, 1 c&s 29.0 mm SL, municipality of Água Fria de Goiás, das Brancas Stream, a tributary of the Tocantizinho River, 14°53'47.2"S, 47°34'58.4"W, 30 June 2014, FF Roxo, GSC Silva, LE Ochoa. LBP 19319, 3, 24.4–28.4 mm SL, municipality of Água Fria de Goiás, das Brancas Stream, tributary of the Tocantizinho River, 14°53'47.2"S, 47°34'58.4"W, 16 August 2014, BF Melo, C Oliveira, GSC Silva, MI Taylor. LBP 19467, 2, 27.6–28.4 mm SL, municipality of Água Fria de Goiás, das Brancas Stream, a tributary of the Tocantizinho River, 14°53'47.2"S, 47°34'58.4"W, 9 November 2014, FF Roxo, GSC Silva, LEO Ochoa, LH Roxo. LBP 19468, 1, 27.7 mm SL, municipality of São João D’Aliança, Roncador Stream, a tributary of das Brancas Stream, 14°43'51.3"S, 47°32'34.0"W, 9 November 2014, FF Roxo, GSC Silva, LE Ochoa, LH Roxo. MZUSP 113919, 2, 21.7–25.0 mm SL, municipality of Água Fria de Goiás, das Brancas Stream, a tributary of the Tocantizinho River, 14°53'47.2"S, 47°34'58.4"W, 27 November 2012, AM Zanata, P Camelier, M Melo, OT Oyakawa.

#### Diagnosis.

Same as for the genus.

#### Description.

Morphometric and meristic data in Table 1. In lateral view, dorsal profile of head strongly convex from snout tip to distal margin of supraoccipital; straight from supraoccipital to dorsal-fin origin; concave and slightly decreasing to end of caudal peduncle. Ventral surface of body, slightly concave at head, straight to convex from posterior end of head to pelvic-fin insertion, and straight but angled to posterior caudal peduncle. Snout tip rounded in dorsal view. Nostril small. Trunk and caudal peduncle rectangular in cross-section. Greatest body depth at dorsal-fin origin. Body progressively narrowing posteriorly from cleithrum to caudal peduncle. Head flat to slightly convex between orbits; superior margin of orbits elevated. Head lacking crests. Head and body plates covered with minute, uniformly sized and evenly distributed odontodes. Head with large area without odontodes around snout. Eye small, situated dorsolaterally just posterior of midpoint.

**Table 1.** Morphometric data for *Microplecostomus forestii* sp. n. SD = standard deviation.

	*Microplecostomus forestii* sp. n., 15 paratypes and the holotype			
	Holotype	Range	Mean	SD
**SL**	38.3	21.7–38.3	27.9	–
**Percentage of SL**				
Predorsal length	45.5	44.5–50.8	47.9	1.8
Head length	34.9	34.5–39.9	37.4	1.5
Head-dorsal length	12.5	10.0–13.5	11.4	1.1
Cleithral width	32.5	31.2–35.2	33.3	1.0
Head-pectoral length	29.7	22.4–32.7	30.1	2.4
Thorax length	19.1	17.1–20.5	19.1	1.0
Pectoral-spine length	19.9	19.2–25.3	21.4	1.7
Abdominal length	21.8	19.4–24.3	21.8	1.2
Pelvic-spine length	20.8	17.0–22.3	20.3	1.6
Post-anal length	34.2	31.8–34.9	33.2	0.9
Anal-fin spine length	12.2	10.6–13.6	12.1	0.8
Dorsal-pectoral distance	25.7	25.3–34.5	28.1	2.2
Dorsal spine length	19.4	18.2–23.0	20.9	1.4
Dorsal-pelvic distance	20.2	16.8–22.3	20.1	1.6
Dorsal-fin base length	18.5	15.1–19.4	17.6	1.2
Caudal peduncle depth	9.5	8.1–10.5	9.6	0.6
Dorsal-anal distance	13.6	13.6–16.8	15.0	1.0
Pelvic-dorsal distance	22.5	20.3–25.7	23.2	1.6
**Percentage of HL**				
Head-eye length	32.3	30.9–36.7	33.9	1.9
Orbital diameter	15.8	13.2–17.2	15.1	1.2
Snout length	61.8	52.9–61.8	57.8	2.8
Internares width	17.0	14.8–19.2	16.7	1.2
Interorbital width	31.5	28.8–34.3	32.1	1.5
Head depth	58.8	55.8–66.6	61.1	2.6
Mouth length	55.8	45.6–66.9	58.7	5.8
Barbel length	4.8	1.2–5.5	3.2	1.2
Dentary tooth cup length	24.2	20.1–27.0	23.3	1.6
Premaxillary tooth cup length	21.7	18.3–25.2	23.3	1.8

**Figure 5.**
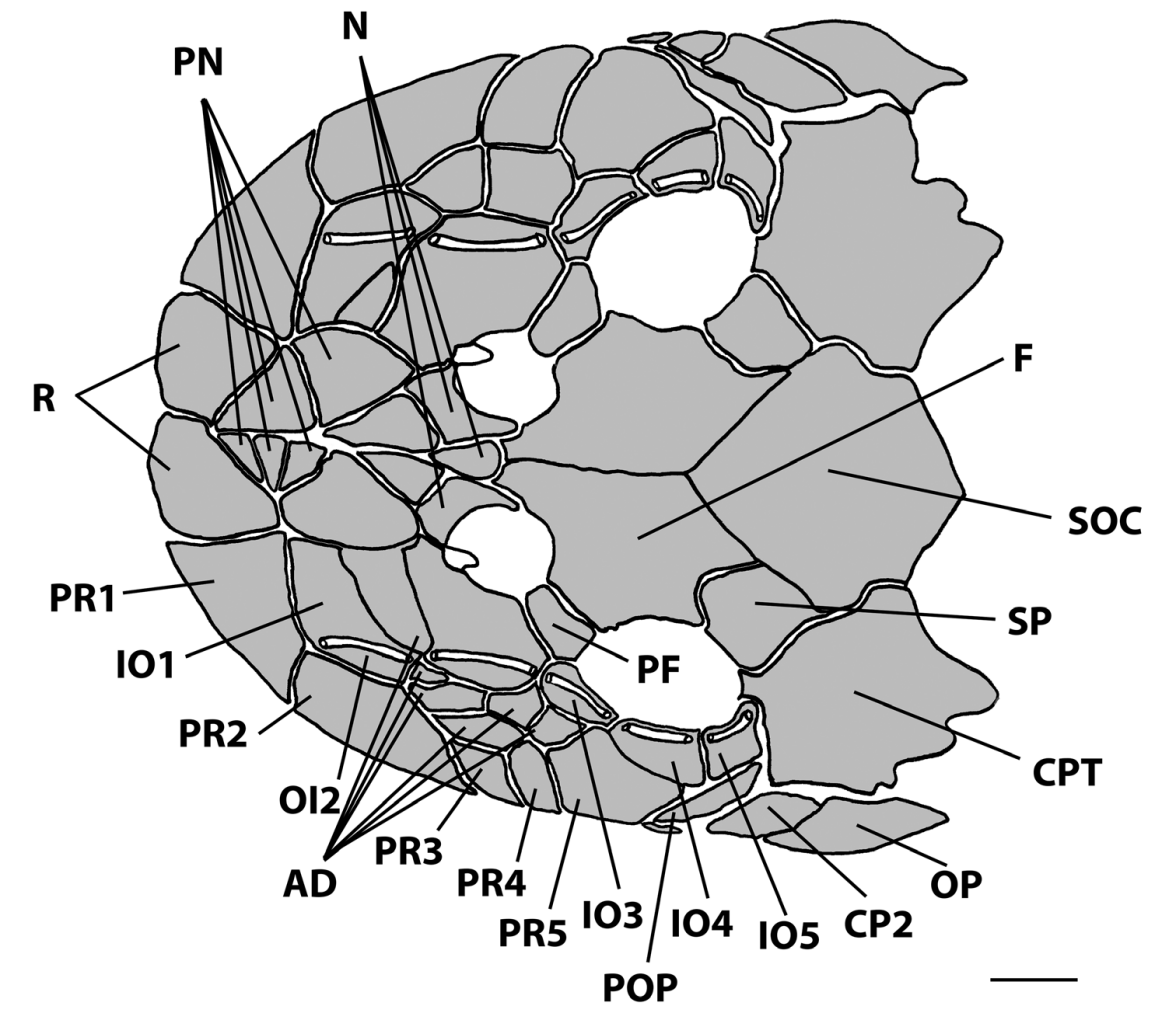
Dorsal view of the head plates in *Microplecostomus forestii* sp. n., LBP 19017, 29.0 mm SL. CPT = compound pterotic; F = frontal; IO1-5 = infraorbitals; N = nasal; OP = opercle; PR1-4 postrostral plates; PF = prefrontal; PN = prenasal; POP = preopercle; R = rostral plate; SP = sphenotic; CP2 = subocular cheek plate; SOC = parieto-supraoccipital; AD = additional plates.

Tip of snout formed by two triangle rostral plates, without odontodes. Nasal plates almost rectangular forming medial nostril margin and contacting pre-nasals anteriorly. Nasal plates posteriorly contacting frontal bones. Lateral margin of head formed by four or five postrostral plates. Complete infraorbital plate series composed of five plates; all infraorbital plates containing latero-sensory canals; first and second infraorbitals largest and third, fourth and fifth smallest. Preopercle elongate, bearing a branch of laterosensory canal. Subocular cheek plates present ventral to preopercle plate. Top of head composed of compound pterotic, supraoccipital, prefrontal, frontal, and sphenotic (Fig. 5); compound pterotic as with fenestrae irregularly distributed and with different sizes and shapes. Anterior margin of mesethmoid pointed and projected anteriorly to condyle.

Lateral ethmoid exposed without odontodes in dorsal view. Lateral ethmoid strut short and broad, nasal capsule partially closed, lateral ethmoid surrounding more than 50% of nasal capsule. Compound pterotic roughly quadrangular, without posterior process, with several fenestrae non-uniform in shape and size. Parieto-supraoccipital not contributing to dorsal portion of swimbladder capsule. Metapterygoid channel present. Hyomandibular square and not sutured to compound pterotic, hyomandibular adductor palatine crest present. Quadrate triangle. Lips large; oral disk rounded and papillose. Premaxillary teeth 44–65 (mode 46). Dentary teeth 45–69 (mode 48). Teeth bicuspid. Maxillar barbel short. Upper pharyngeal tooth-plate small and triangular. Five ceratobranchials with accessory process present and long. Five teeth in ceratobranchial. Four branchiostegal rays.

Dorsal-fin rays II,7; dorsal-fin originating at vertical through posterior end of pelvic-fin base; distal margin slightly convex; dorsal-fin spinelet short and oval in shape. Pectoral-fin rays I,6; distal margin slightly convex; unbranched pectoral-fin ray reaching pelvic-fin origin; unbranched pectoral-fin ray covered with large and pointed odontodes. Pectoral girdle not exposed ventrally. Arrector fossae, partially enclosed by ventral lamina of coracoids, opening relatively large, extending laterally towards base of pectoral fin. Pelvic-fin rays I,5; distal margin of fin slightly convex; tip of adpressed pelvic-fin almost reaching anal-fin origin; unbranched pelvic-fin ray covered with conspicuously pointed, and uniformly distributed odontodes, larger at ventral portion. Pelvic girdle with slender lateropterigium. Basipterygium lacking anterior fenestrae. Anal-fin rays I,5; distal margin slightly convex. Adipose-fin absent. Caudal-fin rays I,7–7,I, truncated with ventral unbranched principal ray longer than dorsal ray.

Compound hypurals 1 and 2 almost completely fused to compound hypurals 3–5, and lower and upper halves fused to last vertebra. Upper and lower lobes of hypural plates of same length. Epural present and separated from hypural plate. Body entirely covered by bony plates, except for ventral surface of head, abdomen and region between compound pterotic and first medial plate. Dorsal series of plates 22–23, mid-dorsal 4–7, median perforated plates 22–23, mid-ventral 11, and ventral 18–20. Trunk with conspicuous, elongated, post-dorsal ridge formed by 14–15 raised, unpaired, median plates; ridge continuous posteriorly with procurrent caudal-fin rays. Six pairs of ribs associated with vertebrae 7–13. Ribs slender and poorly ossified. Total vertebrae 27.

#### Color in life.

**Figure 6.**
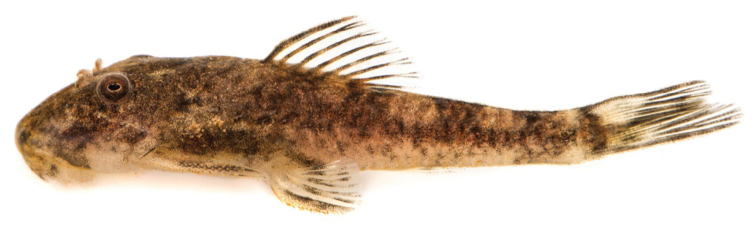
*Microplecostomus forestii* sp. n., live specimen, LBP 19319, paratype, 28.4 mm SL, Tocantins River, Goiás state, Brazil. Photograph: MI Taylor.

Background color of dorsal and ventral surfaces of body yellowish tan. Dorsal surface of head dark brown. Four dark brown saddles on dorsal surface of trunk, most anterior inconspicuous and below dorsal-fin origin, second below end of dorsal-fin, third typically in adipose-fin region, and fourth at end of caudal peduncle. Lateral portion of body with inconspicuous dark stripe from head to caudal fin. Pectoral, pelvic and dorsal fins with three irregular, poorly defined bands. Caudal fin with variegated blotches (Fig. 6).

#### Color in alcohol.

Similar to pattern described for living individuals, but with darker brown color, and darker saddles and stripes (Fig. 1).

#### Sexual dimorphism.

Specimens lacking main sexual dimorphic characters usually present in loricariid species, particularly in Neoplecostominae members, such as (1) a papilla present posteriorly to urogenital opening; (2) an expanded flap skin on dorsal surface of first pelvic-fin ray; and (3) a larger pelvic-fin and body size (all characters present in males), but absent in females. Three hypertrophied bicuspid odontodes are present on lateral portion of body (a characteristic that may be related to mature males), however it is only present in holotype.

#### Etymology.

The specific name, *forestii*, is given in honor of Fausto Foresti, Professor of the university of São Paulo state “Júlio de Mesquita Filho” (Unesp) in Brazil, for his contributions to fish genetics, with more than 250 papers published in this field.

#### Distribution.

**Figure 7.**
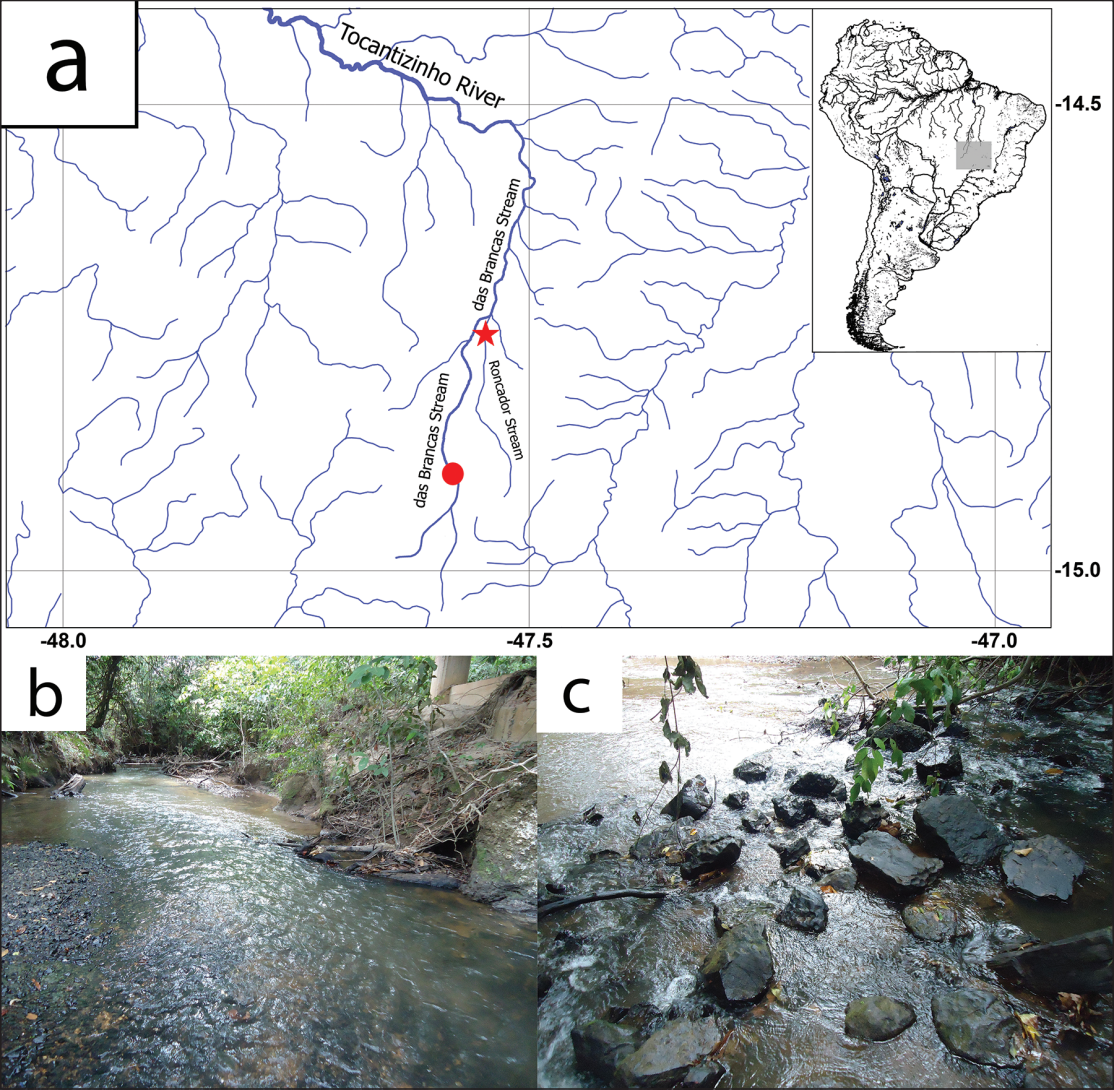
**a** Map showing the distribution of *Microplecostomus forestii* sp. n. Type locality at Roncador Stream, red star – 14°43'51.3"S, 47°32'34.0"W. Paratype locality at das Brancas Stream, red circle – 14°53'47.2"S, 47°34'58.4"W. Habitat where *Microplecostomus forestii* sp. n. is found at **b** Roncador Stream and **c** das Brancas Stream. These are small size streams with a depth of less than 1 m, clear water, the bottom covered with loose stones and shaded margins. Photographs: LH Roxo.

*Microplecostomus forestii* sp. n. is known from two localities, the Roncador Stream and the das Brancas Stream, both tributaries of the Tocantizinho River, in the Tocantins basin (Fig. 7a).

#### Habitat.

*Microplecostomus forestii* sp. n. was collected in shallow, clear waters of about 0.5 m in depth and fast-flowing currents, with an underlying substrate of rock, in areas of flat terrain. The fishes captured were associated with pebbles (Fig. 7b, c). This species is relatively hard to collect and is not abundant. In seven expeditions to the Roncador and das Brancas streams in different periods of the year, we were able to collect only 16 specimens. *Microplecostomus forestii* sp. n. is sympatric with species such as *Creagrutus* sp., *Rhinolekos capetinga* Roxo, Ochoa, Silva & Oliveira, 2015, *Hypostomus* sp., *Phenacorhamdia* sp., *Ancistrus* sp., and *Ituglanis* sp.

##### Sequencing and phylogenetic analysis

The sequences of all 157 specimens are shown in Suppl. material 1 (the same list of species presented by Roxo et al. 2014, but with the inclusion of the voucher and GenBank accession numbers for the specimens of the newly described genus). The concatenated dataset resulted in a matrix of 4,102 base pairs (bps), used in all the phylogenetic and biogeographic analyses, of which 1,361 bps were conserved and 2,657 bps were variable. There was no evidence of saturation in these data, considering that the Iss.c value is higher than the Iss, and the R^2^ value is higher than 0.8 for transitions and transversions, for the concatenated matrix.

**Figure 8.**
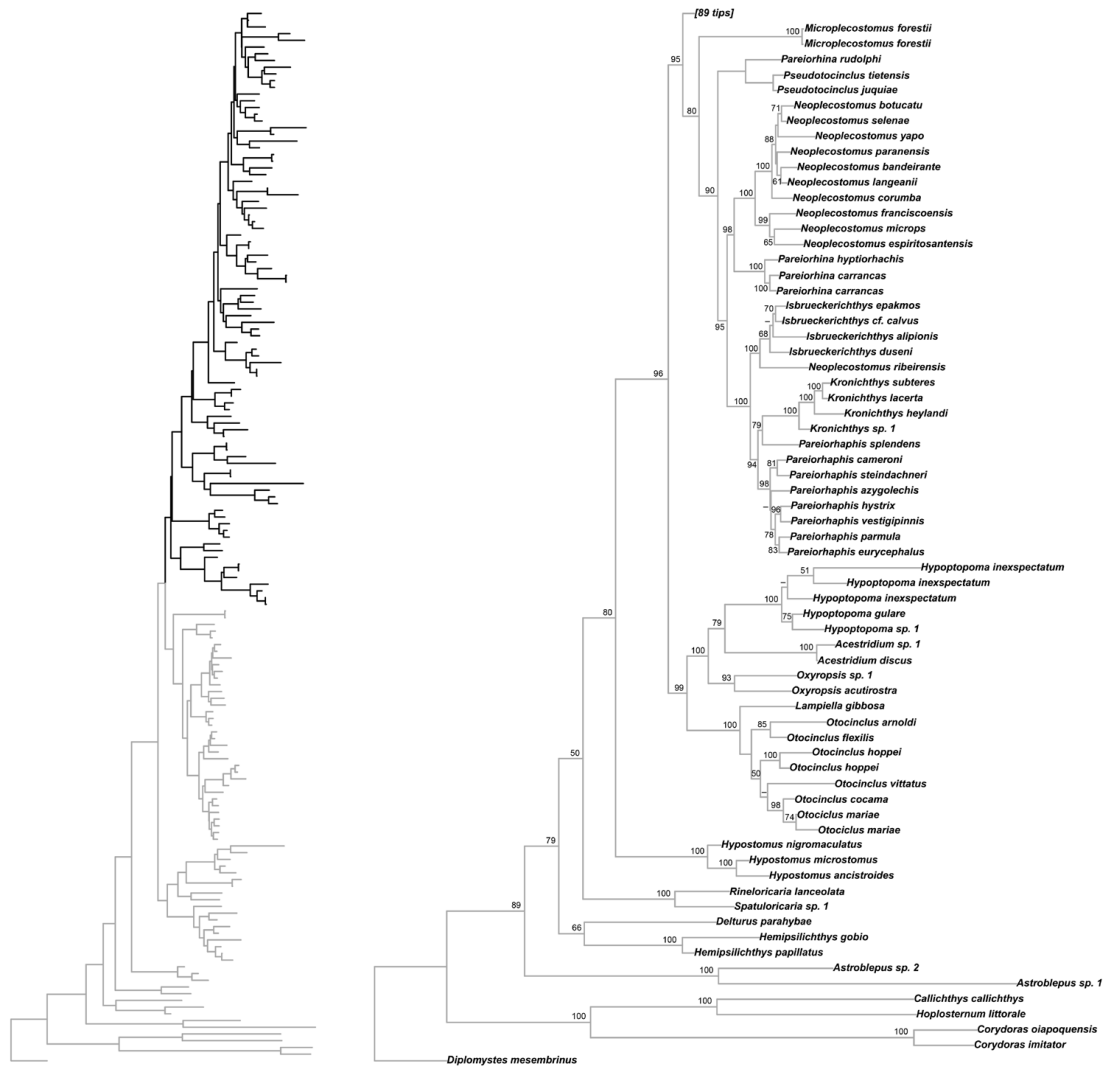
Partial ML tree showing the relationship among the species of the subfamilies Hypoptopomatinae and Neoplecostominae. Numbers above the branches are bootstrap values from 1000 bootstrap pseudoreplicates obtained from the ML analysis. Bootstrap values below 50% (-) are not shown.

**Figure 9.**
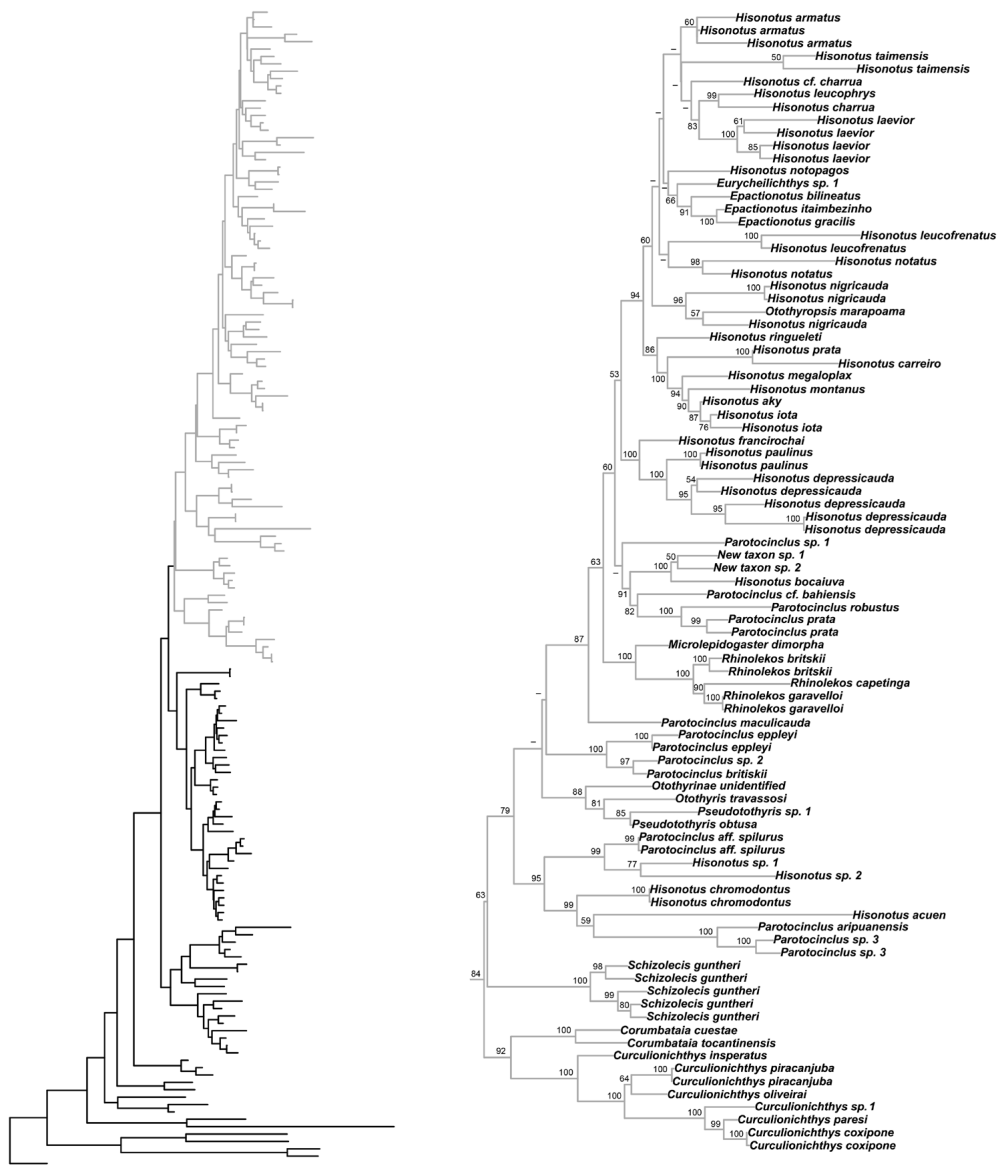
Partial ML tree showing the relationship among the species of the subfamily Otothyrinae. Numbers above the branches are bootstrap values from 1000 bootstrap pseudoreplicates obtained from the ML analysis. Bootstrap values below 50% (-) are not shown.

Our results are very similar to those of of Roxo et al. (2014), in particular, that the Hypoptopomatinae, Neoplecostominae and Otothyrinae clades are monophyletic (Figs 8–9) with strong statistical support (BS = 99 for Hypoptopomatinae; BS = 80 for Neoplecostominae; BS = 84 for Otothyrinae), that the Neoplecostominae is more closely related to the Otothyrinae than to the Hypoptopomatinae (BS = 95), and that these two clades together form the sister group of the Hypoptopomatinae (BS = 96). The new genus *Microplecostomus forestii* sp. n. was placed in the subfamily Neoplecostominae (Fig. 8), forming a sister group with all its members, with strong statistical support (BS = 80).

##### Time calibrated tree and historical biogeography

**Figure 10.**
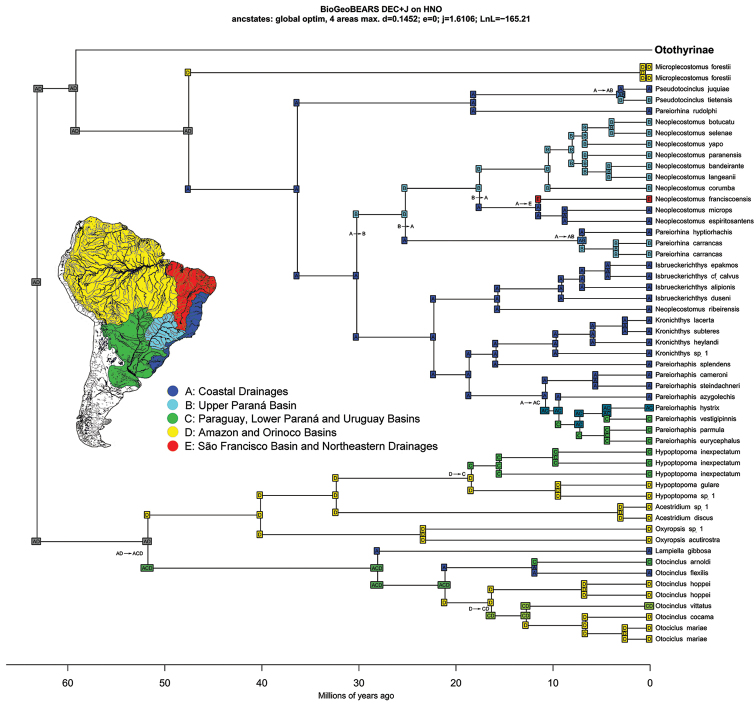
Time-calibrated phylogeny and ancestral range estimates for the Hypoptopomatinae and Neoplecostominae. Divergence ages were calibrated by the origins of the Siluriformes (120 Mya) and the Callichthyidae (55 Mya).

**Figure 11.**
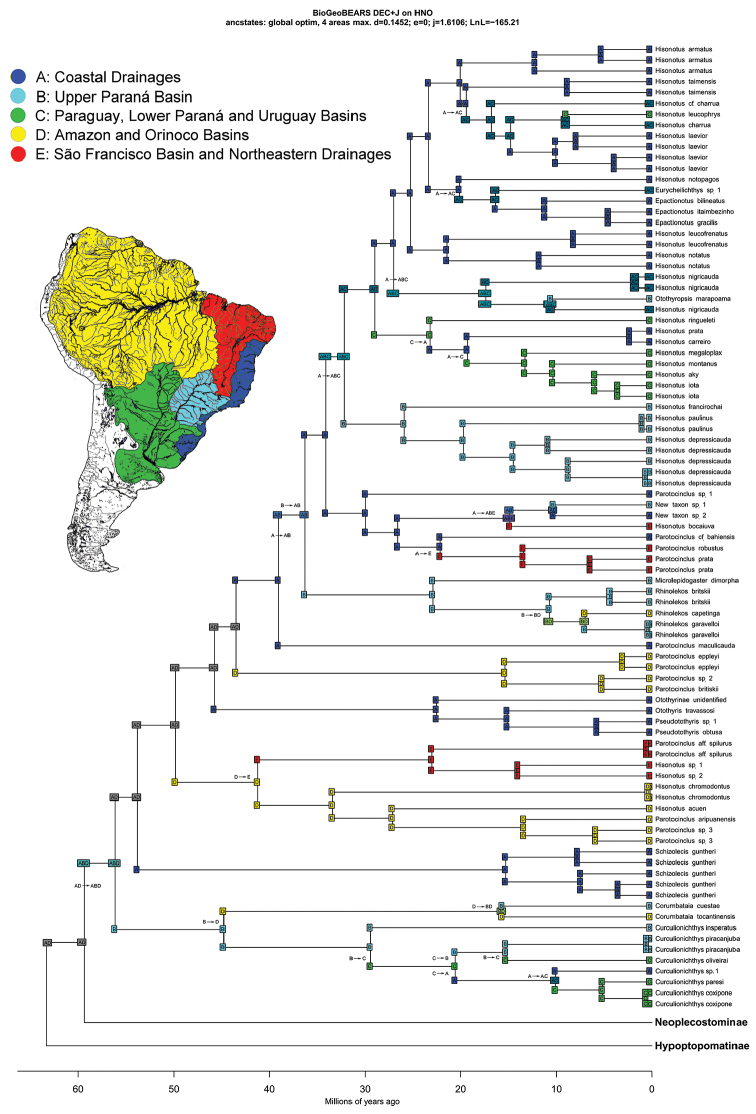
Time-calibrated phylogeny and ancestral range estimates for the Otothyrinae. Divergence ages were calibrated by the origins of the Siluriformes (120 Mya) and the Callichthyidae (55 Mya).

Our time-calibrated tree estimated that the origin of the hypoptopomatine lineage was in the Paleocene, about 63.1 Mya (44.5–83.8 Mya 95% HPD), and is inferred by the DEC+J model to have been located in areas A (Atlantic Coast drainage basins) + D (Amazon and Orinoco basins) (Fig. 10 Region AD). The clade composed of the Neoplecostominae (Fig. 10 Region AD) + Otothyrinae (Fig. 11 Region AD) is estimated by BEAST to have also originated during the Paleocene, about 59.1 Mya (41.4–77.6 Mya 95% HPD), and once again, according to the DEC+J model, in areas A and D. *Microplecostomus forestii* sp. n. is found in the headwaters of the Tocantins River, one of the principal rivers of the Amazon basin (Area D). Our time-calibrated phylogeny estimated that the lineage of the new genus and species arose during the Eocene, 47.5 Mya (32.7–64.5 Mya 95% HPD).

## Discussion

### Taxonomy and phylogenetic relationship

The results of our molecular analyses indicated that *Microplecostomus forestii* sp. n. is the sister-group of all the other members of the Neoplecostominae (Fig. 8), with strong statistical support. *Microplecostomus forestii* sp. n. is distinguished from all other neoplecostomine species by the presence of two autapomorphic characters: (1) three hypertrophied bicuspid odontodes on the lateral portion of the body and (2) a large area without odontodes around the snout, observed in all specimens. Only one (the holotype) of the 16 specimens analyzed presented the three hypertrophied bicuspid odontodes on the lateral portion of the body. We believe that this is a sexually dimorphic character found only in large mature males, although this remains uncertain because the new taxon does not have any other obvious sexual dimorphism, and the trait is only present in the holotype, which cannot be dissected. Sexual dimorphism is very common in other neoplecostomines, in particular species of the genera *Neoplecostomus* (Langeani 1990; Zawadzki et al. 2008; Roxo et al. 2012b) and *Pareiorhaphis* (Pereira and Reis 2002; Pereira 2005). As in the present study, these species have been diagnosed primarily on the basis of the characteristics of the mature males.

Another character used to distinguish *Microplecostomus forestii* sp. n. from other neoplecostomines is the presence of a post-dorsal ridge on the caudal peduncle. Bockmann and Ribeiro (2003) were the first authors to report this character in a neoplecostomine species (*Pareiorhina carrancas*), and Silva et al. (2013) also reported the structure in a second new species of the same genus, *Pareiorhina hyptiorhachis*. This character is also present in species of *Corymbophanes* (Armbruster et al. 2000), a genus assigned to the subfamily Hypostominae, which is found throughout the Potaro River in Guyana, northern South America. Considering that these species are not closely related (Armbruster 2004; Roxo et al. 2014), the presence of a post-dorsal ridge appears to have arisen more than once during the evolution of these species.

The subfamily Neoplecostominae, as defined by Roxo et al. (2014), appears to be monophyletic and forms a sister group to the Otothyrinae, which together form a sister group to the Hypoptopomatinae (this relationship was first reported by Chiachio et al. 2008). The main deviation that we found from the arrangement proposed by Roxo et al. (2014) is in the relationship among the members of the genus *Hisonotus*. In the present study, the species *Hisonotus depressicauda* (Miranda Ribeiro, 1918b), *Hypostomus francirochai* (Ihering, 1928) and *Hypostomus paulinus* (Regan, 1908) appeared as a monophyletic group which includes most of the *Hisonotus* species found in southern Brazil, the type species of *Hisonotus* (i.e. *Hisonotus notatus* Eigenmann & Eigenmann, 1889), *Otothyropsis marapoama* Ribeiro, Carvalho & Melo, 2005, *Eurycheilichthys* and *Epactionotus*. In Roxo et al. (2014) *Hisonotus depressicauda*, *Hypostomus francirochai* and *Hypostomus paulinus* were not closely related to the former species, but formed a clade with *Parotocinclus* and *Hisonotus* species from the São Francisco River basin, albeit with reduced statistical support (see Fig. 4 in Roxo et al. 2014).

### Historical biogeography

Using the DEC model to estimate ancestral species ranges, Roxo et al. (2014) suggested that the ancestral lineages of the Hypoptopomatinae, Neoplecostominae and Otothyrinae subfamilies (the HNO-Clade) originated on the Atlantic Coast of southeastern Brazil (area A, see Fig. 5 in Roxo et al. 2014). However, in a geographic area dominated by headwater capture events, ancestral lineages would be expected to be more widely distributed in adjacent hydrographic systems (see e.g. Albert et al. 2011). Given this, Roxo et al. (2014) did not reject the hypothesis that the ancestral lineages of the HNO-clade were more widely distributed in South America during the early Cenozoic, including much of the modern Atlantic Coast (area A), upper Paraná (area B), Paraguay, lower Paraná and Uruguay (area C), and Amazon and Orinoco basins (area D).

Our ancestral range estimates found, using the DECj model and including *Microplecostomus forestii* sp. n. in the HNO phylogeny, that the ancestral lineages of these three subfamilies were widely distributed on the Atlantic Coast (area A) and in the Amazon and Orinoco basins (area D). While these two areas are not adjacent (i.e. they do not share an endpoint or border), a number of studies have found evidence of the historical mixing of the faunas of the headwaters of the Amazon and Paraná rivers, and the drainage basins of the Atlantic Coast. The historical connection between the Paraguay and Amazon basins has been known for more than a century (e.g. Eigenmann and Eigenmann 1891; Jordan 1896; Eigenmann 1906; Pearson 1937; Carvalho and Albert 2011; Ribeiro et al. 2013), and may account for the geodispersal (*sensu* Albert and Reis 2011) from the Amazon drainage basins (in particular the Madeira, Tocantins and Xingu) located on the Brazilian Shield. Even so, geodispersal events in the reverse direction, i.e., from south to north should also be expected (Roxo et al. 2014), and the dispersal of the hypoptopomatine lineages (*sensu* Chiachio et al. 2008) is considered to be the result of historical connections among the Amazon, Orinoco and Paraguay basins (Albert et al. 2011; Roxo et al. 2014). A number of authors have proposed headwater capture as the likely mechanism determining the distribution of ancestral fish lineages in the Tietê, Paraíba do Sul, São Francisco and Ribeira de Iguape basins on the Brazilian Shield (Ab’Saber 1957; Ab’Saber 1998; Ribeiro 2006; Roxo et al. 2012c; 2014). The historical dispersal of ancestral fish lineages between areas A and D is thus quite conceivable.

All neoplecostomine lineages are found in southern and southeastern of South America, except *Microplecostomus forestii* sp. n. and *Pareiorhaphis regani* (Giltay, 1936), which are from the Amazon basin (area D). In a paper on the evolution of plants, Stebbins (1974) discussed the concepts of evolutionary museum and evolutionary cradle, which are used to define species occurrence patterns within an area. An evolutionary cradle is defined as an area of high speciation rates, while an evolutionary museum is an area with low extinction rates, where environmental conditions combine to preserve lineages over long periods of time. Roxo et al. (2014) suggested that in the Hypoptopomatinae, the *Lampiella gibbosa* (Miranda Ribeiro, 1908) and *Otocinclus affinis* (Steindachner, 1877b) lineages are relicts of the Atlantic Coast drainage basins, considering that other *Otocinclus* species are widely distributed in the lowland Amazon and Paraná-Paraguay basins. The new genus and species described here, *Microplecostomus forestii* sp. n., also appears to be a relict lineage of the Tocantins River basin (Amazon basin), given that all other neoplecostomine species, except *Pareiorhaphis regani*, are present in the upper Paraná, lower Paraná-Paraguay, and coastal drainage basins of the Brazilian Shield.

### Comparative material

*Chauliocheilos saxatilis* Martins, Andrade, Rosa & Langeani, 2014: paratype, MZUSP 114758, 2, 38.9–40.2 mm SL, municipality of Itamarandiba, Minas Gerais state, Brazil, tributary of the Itamarandiba River.

*Curculionichthys insperatus* Britski & Garavello, 2003: LBP 4945, 5, 27.3−28.5 mm SL, 2 c&s, 28.2−29.9 mm SL, municipality of Botucatu, São Paulo state, Brazil, Tietê River basin.

*Gymnotocinclus anosteos* Carvalho, Lehmann A. & Reis, 2008: LBP 17125, 3, 18.8–33.0 mm SL, municipality of Alto Paraíso de Goiás, Goiás state, Brazil, Tocantins River basin.

*Hisonotus acuen* Silva, Roxo & Oliveria, 2014: holotype, MZUSP 115350, 25.9 mm SL, municipality of Querência, Mato Grosso state, Brazil, Xingu River basin; paratype, LBP 15755, 16, 19.5–26.0 mm SL, municipality of Ribeirão Cascalheira, Mato Grosso, Xingu basin.

*Hisonotus bocaiuva* Roxo, Silva, Oliveira & Zawadzki, 2013: holotype, MZUSP 112204, 24.2 mm SL, municipality of Bocaiúva, Minas Gerais state, Brazil, São Francisco River basin; paratype, LBP 9817, 9, 3 c&s, 18.3−23.2 mm SL, municipality of Bocaiúva, Minas Gerais state, Brazil São Francisco River basin.

*Hisonotus notatus* Eigenmann & Eigenmann, 1889a: LBP 18472, 7, 30.1–38.3 mm SL, municipality of Silva Jardim, Rio de Janeiro state, Brazil, coastal drainage basin.

*Isbrueckerichthys alipionis* (Gosline, 1947): LBP 7373, 17, 31.7–81.6 mm SL, municipality of Iporanga, São Paulo state, Brazil, Ribeira de Iguape River basin;

*Kronichthys subteres* Miranda Ribeiro 1908: LBP 515, 31, 28.4–61.9 mm SL, municipality of Iporanga, São Paulo state, Brazil, Ribeira de Iguape River basin.

*Lampiella gibbosa* (Miranda Ribeiro, 1908): LBP 7430, 5, 25.6−26.1 mm SL, municipality of Jacupiranga, São Paulo state, Brazil, Ribeira de Iguape River basin.

*Microlepidogaster arachas* Martins, Calegari & Langeani, 2013: LBP 10882, 3, 22.8−35.3 mm SL, municipality of Araxás, Minas Gerais state, Brazil, Paraná River basin.

*Nannoplecostomus eleonorae* Ribeiro, Lima & Pereira, 2012: LBP 19016, 51, 19.9–25.4 mm SL, municipality of Guarani de Goiás, Goiás state, Brazil, Tocantins River basin.

*Neoplecostomus microps* (Steindachner, 1877a): LBP 8036, 38, 41.3–65.0 mm SL, municipality of Piquete, São Paulo state, Brazil, Paraíba do Sul River basin.

*Neoplecostomus franciscoensis* Langeani, 1990: LBP 6489, 50, 42.8–55.9 mm SL, municipality of São Bartolomeu, Minas Gerais state, Brazil, São Francisco River basin.

*Neoplecostomus paranensis* Langeani, 1990: holotype, MZUSP 38572, 71.4 mm SL, municipality of Cajuru, Minas Gerais state, Brazil, Grande River basin.

*Otocinclus affinis* (Steindachner, 1877b): 19, 19.5−28.9 mm SL, municipality of Poconé, Mato Grosso state, Brazil, Paraguay River basin.

*Otocinclus vittatus* Regan, 1904: 27, 18.2−21.7 mm SL, municipality of Cáceres, Mato Grosso state, Brazil, Paraguay River basin.

*Otothyropsis marapoama* Ribeiro, Carvalho & Melo, 2005: LBP 4698, 6, 23.9−36.3 mm SL, municipality of Marapoama, São Paulo state, Brazil, Tietê River basin.

*Pareiorhaphis splendens* (Bizerril, 1995): LBP 1117, 20, 32.0–100.0 mm SL, municipality of Morretes, Paraná state, Brazil, Atlantic Coast drainage basins.

*Pareiorhaphis steindachneri* (Miranda Ribeiro, 1918a): LBP 739, 6, 33.8–49.0 mm SL, municipality of Jaraguá do Sul, Santa Catarina state, Brazil, Atlantic Coast drainage basins.

*Pareiorhina brachyrhyncha* Chamon, Aranda & Buckup, 2005: LBP 12240, 50, 26.4–36.9 mm SL, municipality of Pindamonhangaba, São Paulo state, Brazil, Paraíba do Sul River basin.

*Pareiorhina cepta* Roxo, Silva, Mehanna & Oliveira, 2012d: holotype, MZUSP 111095, 41.5 mm SL, municipality of São Roque de Minas, Minas Gerais state, Brazil, São Francisco basin, paratypes, LBP 10287, 13, 21.5–43.6 mm SL, municipality of São Roque de Minas, Minas Gerais, Brazil, Paraíba do Sul River basin.

*Pareiorhina rudolphi* (Miranda Ribeiro, 1911): LBP 8044, 18, 31.7–48.9 mm SL, municipality of Piquete, São Paulo state, Brazil, Paraíba do Sul River basin.

*Parotocinclus maculicauda* (Steindachner, 1877b): LBP 2869, 15, 20.2−44.7 mm SL, municipality of Miracatu, São Paulo state, Brazil, Ribeira de Iguape River basin.

*Plesioptopoma curvidens* Reis, Pereira & Lehmann A, 2012: LBP 17394, 39, 26.1–81.7 mm SL, municipality of Cristiano Otoni, Minas Gerais state, Brazil, São Francisco River basin.

*Pseudotocinclus juquiae* Takako, Oliveira & Oyakawa, 2005: LBP1081, 2, 29.0–31.9 mm SL, municipality of Juquitiba, São Paulo state, Brazil, Atlantic Coast drainage basins.

*Pseudotocinclus tietensis* (Ihering, 1907): LBP 2931, 3, 38.6–62.3 mm SL, municipality of Salesópolis, São Paulo state, Brazil, Tietê River basin.

*Schizolecis guntheri* (Miranda Ribeiro, 1918b): LBP 14335, 18, 18.3–35.3 mm SL, municipality of São Sebastião, São Paulo state, Brazil, Atlantic Coast drainage basins.

## Supplementary Material

XML Treatment for Microplecostomus

XML Treatment for Microplecostomus forestii

## Supplementary materials

Supplementary material 1 Table S1 Data type: Microsoft Word document Explanation note: Species included in the present study with voulchers and GenBank accession numbers. This dataset is made available under the Open Database License (http://opendatacommons.org/licenses/odbl/1.0/). The Open Database License (ODbL) is a license agreement intended to allow users to freely share, modify, and use this Dataset while maintaining this same freedom for others, provided that the original source and author(s) are credited.Gabriel S. C. Silva, Fábio F. Roxo, Luz E. Ochoa, Claudio Oliveira

Supplementary material 2 Table S2 Data type: Microsoft Word document Explanation note: Models tested to estimate distribution ranges inherited by the descending lineages at each node of the tree. This dataset is made available under the Open Database License (http://opendatacommons.org/licenses/odbl/1.0/). The Open Database License (ODbL) is a license agreement intended to allow users to freely share, modify, and use this Dataset while maintaining this same freedom for others, provided that the original source and author(s) are credited.Gabriel S. C. Silva, Fábio F. Roxo, Luz E. Ochoa, Claudio Oliveira
