# Supplementary material for: Description of a new catfish genus (Siluriformes, Loricariidae) from the Tocantins River basin in central Brazil, with comments on the historical zoogeography of the new taxon
Source: Zookeys. 2016 Jun 14;(598):129–57. doi: 10.3897/zookeys.598.7400 (PMC4926676; doi:10.3897/zookeys.598.7400)
Supplement: Supplementary material 1 — Table S1 [file zookeys-598-129-s001.doc]

**Supplementary table 1.** Species included in the present study. ANSP = Academy of Natural Sciences of Drexel University, Philadelphia; AUM = Auburn University Natural History Museum; LBP = Laboratório de Biologia e Genética de Peixes, Universidade Estadual Paulista; MCP = Museu de Ciências e Tecnologia, Pontifícia Universidade Católica do Rio Grande do Sul; MNRJ = Museu Nacional da Universidade Federal do Rio de Janeiro; NUP = Núcleo de Pesquisas em Limnologia, Ictiologia e Aquicultura, Universidade Estadual de Maringá; MHNG = Museum of Natural History of the City of Geneva.

| Number | Collection No | Fish No | GenBank (Reticulon/16S/COI/CytB) | Species | Location (river, city, state, country) |
| --- | --- | --- | --- | --- | --- |
| 1 | LBP 6037 | 29054 | KM205118/KM104327/JN998540/KM104515 | *Hisonotus armatus* | Rio Maquiné/Osório/RS/Brazil |
| 2 | MCP 21375 |  | -/-/EU371011/- | *Hisonotus armatus* | Lagoa dos Patos/São José do Norte/RS/Brazil |
| 3 | MCP 37682 |  | KM205119/KM104328/KM104432/KM104516 | *Hisonotus armatus* | Arroio Arambare/Pedro Osório/RS/Brazil |
| 4 | LBP 3472 | 20258 | KM205120/KM104329/JN998579/KM104517 | *Hisonotus notatus* | Afluente Rio Aduelas/Macaé/RJ/Brazil |
| 5 | MNRJ 37474 |  | KM205121/KM104330/JN998532/KM104518 | *Hisonotus notatus* | -/Casimiro de Abreu/RJ/Brazil |
| 6 | LBP 4765 | 25554 | KM205122/KM104331/KM104433/- | *Hisonotus taimensis* | Rio Guaíba/Barra do Ribeiro/RS/Brazil |
| 7 | LBP 13147 | 51066 | -/KM104332/KM104434/KM104519 | *Hisonotus taimensis* | Riacho sem nome/Agudo/RS/Brazil |
| 8 | LBP 7407 | 35655 | -/KM104333/KM104435/- | *Hisonotus leucofrenatus* | Rio Batatau/Itapeúna/SP/Brazil |
| 9 | MCP 31819 |  | KM205123/KM104334/JN998552/KM104520 | *Hisonotus leucofrenatus* | Afluente Rio Nhundiaquara/Morretes/PR/Brazil |
| 10 | MCP 41351 |  | KM205124/KM104335/JN998546/KM104521 | *Hisonotus leucophrys* | Rio Ariranhas/Xavantina/SC/Brazil |
| 11 | MCP 21644 |  | KM205125/KM104336/JN998529/KM104522 | *Hisonotus charrua* | Rio Quaraí Mirim/Quaraí/RS/Brazil |
| 12 | MCP 37684 |  | KM205126/KM104337/JN998535/KM104523 | *Hisonotus laevior* | Arroio Arambare/Pedro Osório/ RS/Brazil |
| 13 | LBP 13187 | 51070 | KM205127/KM104338/JQ015384/KM104524 | *Hisonotus laevior* | Riacho sem nome/Camaquá/RS/Brazil |
| 14 | LBP 3376 | 21246 | KM205128/KM104339/JN998536/KM104525 | *Hisonotus laevior* | Arroio dos Corrientes/Pelotas/RS/Brazil |
| 15 | LBP 3376 | 21248 | KM205129/KM104340/JN998538/- | *Hisonotus laevior* | Arroio dos Corrientes/Pelotas/RS/Brazil |
| 16 | LBP 4720 | 24941 | KM205130/KM104341/JN998531/- | *Hisonotus* cf. *charrua* | Arroio Cuaró Grande/Artigas/Uruguay |
| 17 | MCP 40762 |  | -/KM104342/KM104436/KM104526 | *Hisonotus notopagos* | Arroio Mantiqueira/Lavras do Sul/RS/Brazil |
| 18 | LBP 4723 | 24951 | KM205131/KM104343/EU817529/KM104527 | *Eurycheilichthys* sp. 1 | Arroio Jaboticaba/Veranópolis/RS/Brazil |
| 19 | LBP 4871 | 24919 | KM205132/KM104344/EU817532/KM104528 | *Epactionotus bilineatus* | Arroio Carvalho/Itati/RS/Brazil |
| 20 | MCP 23683 |  | -/-/EU371004/- | *Epactionotus itaimbezinho* | Rio Mangue/Morrinho do Sul/RS/Brazil |
| 21 | MCP 23606 |  | -/-/EU371005/- | *Epactionotus gracilis* | Rio Morto/Meleiro/SC/Brazil |
| 22 | LBP 3335 | 20465 | -/KM104345/JN998575/KM104529 | *Hisonotus nigricauda* | Arroio sem nome/Rio Grande/RS/Brazil |
| 23 | LBP 579 | 8565 | KM205133/KM104346/KM104437/KM104530 | *Hisonotus nigricauda* | Afluente Rio Guaíba/Eldorado do Sul/RS/Brazil |
| 24 | LBP 4719 | 25636 | KM205134/KM104347/EU817522/- | *Hisonotus nigricauda* | Arroio do Nariz/Rosário do Sul/RS/Brazil |
| 25 | LBP 5528 | 25343 | KM205135/KM104348/EU817534/KM104531 | *Otothyropsis marapoama* | Ribeirão Cubatão/Marapoama/SP/Brazil |
| 26 | LBP 4863 | 24930 | KM205136/KM104349/KM104438/KM104532 | *Hisonotus ringueleti* | Arroio Cuaró Grande/Artigas/Uruguay |
| 27 | MCP 40943 |  | -/KM104350/KM104439/- | *Hisonotus carreiro* | Arroio Guabiju/Guabiju/RS/Brazil |
| 28 | MCP 40942 |  | -/KM104351/KM104440/KM104533 | *Hisonotus prata* | Rio da Prata/Nova Prata/RS/Brazil |
| 29 | MCP 41352 |  | KM205137/KM104352/JN998543/KM104534 | *Hisonotus megaloplax* | Arroio Cragoata/Passo Fundo/RS/Brazil |
| 30 | LBP 13055 | 51035 | -/KM104353/KM104441/- | *Hisonotus montanus* | Rio Canoas/Vargem/SC/Uruguay |
| 31 | MCP 41474 |  | -/KM104354/JN998533/KM104535 | *Hisonotus aky* | Rio Forquilha/Paim Filho/RS/Brazil |
| 32 | LBP 13072 | 51046 | KM205138/KM104355/JQ015387/- | *Hisonotus iota* | Rio Chapecó/Coronel Freitas/SC/Brazil |
| 33 | MCP 40029 |  | KM205139/KM104356/JN998544/KM104536 | *Hisonotus iota* | Rio Chapeco/Coronel Freitas/SC/Brazil |
| 34 | LBP 8249 | 38464 | -/KM104357/KM104442/KM104537 | *Parotocinclus prata* | Rio Santo Antônio/Luizlândia/MG/Brazil |
| 35 | LBP 7472 | 32387 | JN689284/KM104358/JN089798/JN089773 | *Parotocinclus prata* | Córrego Guarda-Mor/Guarda-Mor/MG/Brazil |
| 36 | LBP 8258 | 38298 | -/-/KM104443/KM104538 | *Parotocinclus robustus* | Córrego Cachoeira/Bocaiúva/MG/Brazil |
| 37 | LBP 7182 | 34694 | KM205140/KM104359/KM104444/KM104539 | *Parotocinclus* cf. *bahiensis* | Rio Coité/Lençóis/BA/Brazil |
| 38 | LBP 5867 | 28345 | KM205141/KM104360/KM104445/- | *New taxon* 1 | Rio Curral das Éguas/Três Marias/MG/Brazil |
| 39 | LBP 17402 | 67143 | -/KM104361/KM104446/KM104540 | *Hisonotus bocaiuva* | Córrego Cachoeira/Bocaiuva/MG/Brazil |
| 40 | LBP 7244 | 33302 | KM205142/KM104362/KM104447/KM104541 | *New taxon* 2 | Afluente Rio Araguari/Perdizes/MG/Brazil |
| 41 | LBP 3457 | 20293 | -/KM104363/KM104448/KM104542 | *Parotocinclus* sp. 1 | Rio da Conceição/Guarapari/ES/Brazil |
| 42 | LBP 13923 | 58218 | KM205143/KM104364/KM104449/KM104543 | *Hisonotus francirochai* | Córrego sem nome/Capitinga/MG/Brazil |
| 43 | LBP 871 | 8801 | KM205144/KM104365/KM104450/KM104544 | *Hisonotus paulinus* | Afluente do rio Grande/Paranapiacaba, SP/Brazil |
| 44 | LBP 3932 | 22661 | KM205145/KM104366/KM104451/KM104545 | *Hisonotus paulinus* | Rio Paraitinguinha/Salesópolis/SP/Brazil |
| 45 | LBP 17583 | 69477 | -/KM104367/KM104452/KM104546 | *Hisonotus depressicauda* | Riacho sem nome/Sapucaí Mirim/MG/Brazil |
| 46 | LBP 17583 | 69479 | -/KM104368/KM104453/KM104547 | *Hisonotus depressicauda* | Riacho sem nome/Sapucaí Mirim/MG/Brazil |
| 47 | LBP 8051 | 37802 | KM205146/KM104369/KM104454/KM104548 | *Hisonotus depressicauda* | Ribeirão do Salto/Delfim Moreira/MG/Brazil |
| 48 | MCP 22602 |  | -/KM104370/KM104455/KM104549 | *Hisonotus depressicauda* | Arroio Barra Grande/Paulo Frontin/PR/Brazil |
| 49 | LBP 2910 | 18756 | -/KM104371/KM104456/KM104550 | *Hisonotus depressicauda* | Ribeirão da Quinta/Botucatu/SP/Brazil |
| 50 | LBP 4854 | 25626 | EU817533/KM104372/KM104457/- | *Microlepidogaster dimorpha* | Riacho Grotão/Araxá/MG/Brazil |
| 51 | LBP 7253 | 33309 | KM205147/KM104373/KM104458/- | *Rhinolekos britskii* | Córrego sem nome/Pires do Rio/GO/Brazil |
| 52 | LBP 7245 | 34405 | KM205148/KM104374/KM104459/KM104551 | *Rhinolekos britskii* | Afluente Rio Arapuca/Bela Vista de Goias/GO/Brazil |
| 53 | LBP 5848 | 28365 | KM205149/KM104375/KM104460/- | *Rhinolekos capetinga* | Riacho sem nome/Águas Fria de Goiás/GO/Brazil |
| 54 | LBP 7246 | 34410 | KM205150/KM104376/KM104461/- | *Rhinolekos garavelloi* | Córrego Fazenda Lageado/Caldas Novas/GO/Brazil |
| 55 | LBP 7246 | 33305 | KM205151/KM104377/KM104462/- | *Rhinolekos garavelloi* | Córrego Fazenda Lageado/Caldas Novas/GO/Brazil |
| 56 | LBP 2869 | 18571 | EU817527/KM104378/KM104463/KM104552 | *Parotocinclus maculicauda* | Rio Fau/Miracatu/SP/Brazil |
| 57 | LBP 4722 | 24946 | EU817525/KM104379/KM104464/- | *Pseudotothyris* sp. 1 | Lago Acaraí/São Francisco do Sul/SC/Brazil |
| 58 | LBP 2094 | 13851 | KM205152/KM104380/KM104465/- | *Pseudotothyris obtusa* | Riacho Descoberto/Guaratuba/PR/Brazil |
| 59 | LBP 1971 | 13685 | EU817526/KM104381/KM104466/KM104553 | *Otothyris travassosi* | Riacho Rosário/Canavieiras/BA/Brazil |
| 60 | MHNG 2586.95 | BR1200 | EU817552/-/-/- | *Otothyrinae unidentified* | Rio Taiaçupeba/-/SP/Brazil |
| 61 | LBP 6950 | 35328 | KM205153/KM104382/KM104467/- | *Parotocinclus* sp. 2 | Igarapé Nouba Uba/São Gabriel da Cachoeira/AM/Brazil |
| 62 | ANSP 179131 |  | -/-/GQ225426/- | *Parotocinclus britskii* | -/-/-/-/ |
| 63 | LBP 4787 | 25579 | EU817528/KM104383/KM104468/KM104554 | *Parotocinclus eppleyi* | Caño Tama Tama/-/-/Venezuela |
| 64 | AUM 43947 |  | -/-/EU359455/- | *Parotocinclus eppleyi* | -/-/-/-/ |
| 65 | LBP 5624 | 35327 | KM205154/KM104384/KM104469/- | *Parotocinclus* aff. *spilurus* | Rio Maravilha/Balsas/MA/Brazil |
| 66 | LBP 5624 | 35326 | KM205155/KM104385/KM104470/- | *Parotocinclus* aff. *spilurus* | Rio Maravilha/Balsas/MA/Brazil |
| 67 | LBP 334 | 4276 | KM205156/KM104386/KM104471/KM104555 | *Hisonotus* sp. 1 | Ribeirão Santo Inácio/Moema/MG/Brazil |
| 68 | LBP 8276 | 38487 | KM205157/KM104387/KM104472/KM104556 | *Hisonotus* sp. 2 | Rio Verde Grande/Jaíba/MG/Brazil |
| 69 | LBP 16277 | 61771 | KM205158/KM104388/KM104473/KM104557 | *Hisonotus acuen* | Rio Feio/Querência/MT/Brazil |
| 70 | LBP 7948 | 35750 | KM205159/KM104389/KM104474/- | *Hisonotus chromodontus* | Rio dos Patos/Nova Mutum/MT/Brazil |
| 71 | LBP 7948 | 35749 | KM205160/KM104390/KM104475/- | *Hisonotus chromodontus* | Rio dos Patos/Nova Mutum/MT/Brazil |
| 72 | LBP 10981 | 50459 | -/KM104391/KM104476/KM104558 | *Parotocinclus aripuanensis* | Rio Lajeado/Guajará Mirim/RO/Brazil |
| 73 | LBP 1572 | 11537 | -/KM104392/KM104477/KM104559 | *Parotocinclus* sp. 3 | Ribeirão Ínsula/Barra do Garça/MT/Brazil |
| 74 | LBP 2414 | 16276 | KM205161/KM104393/KM104478/KM104560 | *Parotocinclus* sp. 3 | Córrego Fundo/Barra do Garça/MT/Brazil |
| 75 | LBP 2514 | 13846 | EU817539/KM104394/KM104479/- | *Schizolecis guntheri* | Rio Sagrado/Morretes/PR/Brazil |
| 76 | LBP 3238 | 19471 | EU817536/KM104395/KM104480/- | *Schizolecis guntheri* | Rio Garuva/Garuva/SC/Brazil |
| 77 | LBP 2513 | 15240 | EU817535/KM104396/KM104481/- | *Schizolecis guntheri* | Rio Macacu/Itaboraí/RJ/Brazil |
| 78 | LBP 2401 | 15272 | EU817538/KM104397/KM104482/- | *Schizolecis guntheri* | Rio São Pedro/-/RJ/Brazil |
| 79 | LBP 2988 | 19646 | EU817537/KM104398/KM104483/KM104561 | *Schizolecis guntheri* | Rio Indaiá/Ubatuba/SP/Brazil |
| 80 | LBP 1653 | 11477 | EU817520/KM104399/KM104484/- | *Corumbataia tocantinensis* | Rio Vermelho/Goias/GO/Brazil |
| 81 | LBP 2001 | 12191 | EU817521/KM104400/FJ965499/FJ965509 | *Corumbataia cuestae* | Rio Alambari/Botucatu/SP/Brazil |
| 82 | LBP 5529 | 25381 | EU817518/KM104401/KM104485/KM104562 | *Curculionichthys insperatus* | Ribeirão Cubatão/ Marapoama/SP/Brazil |
| 83 | LBP 1325 | 11238 | KM205162/KM104402/KM104486/KM104563 | *Curculionichthys oliveirai* | Ribeirão Keller/Marialva/PR/Brazil |
| 84 | LBP 17256 | 66651 | KM205163/KM104403/KM104487/KM104564 | *Curculionichthys piracanjuba* | Córrego sem nome/Morrinhos/GO/Brazil |
| 85 | LBP 17256 | 66655 | KM205164/KM104404/KM104488/KM104565 | *Curculionichthys piracanjuba* | Córrego sem nome/Morrinhos/GO/Brazil |
| 86 | MNRJ 29502 | 490 | -/KM104405/KM104489/KM104566 | *Curculionichthys* sp. 3 | -/Cotriguaçu/MT/Brazil |
| 87 | LBP 17532 | 68706 | -/KM104406/KM104490/KM104567 | *Curculionichthys paresi* | Rio Maracanã/Tangará da Serra/MT/Brazil |
| 88 | LBP 5062 | 26057 | KM205165/KM104407/KM104491/- | *Curculionichthys coxipone* | Afluente Rio Aricá Mirim/Chapada dos Guimarães/MT/Brazil |
| 89 | LBP 5062 | 26056 | KM205166/KM104408/KM104492/- | *Curculionichthys coxipone* | Afluente Rio Aricá Mirim/Chapada dos Guimarães/MT/Brazil |
| 90 | LBP 17318 | 66664 | -/KX355621/-/KX355623 | *Microplecostomus forestii* | Córrego Roncador/São João da Aliança/GO/Brazil |
| 91 | LBP 17318 | 66665 | -/KX355622/-/KX355624 | *Microplecostomus forestii* | Córrego Roncador/São João da Aliança/GO/Brazil |
| 92 | LBP 2931 | 18994 | JN689285/JN089823/JN089799/JN089774 | *Pseudotocinclus tietensis* | Rio Paraitinga/Salesópolis/SP/Rio Tietê Basin |
| 93 | LBP 616 | 7564 | JN689286/FJ625810/FJ625819/FJ965511 | *Pseudotocinclus juquiae* | Rio Juquiá/Juquitiba/SP/Rio Ribeira do Iguape Basin |
| 94 | LBP 4391 | 24189 | JN689280/ FJ434517/FJ434540/FJ965510 | *Pareiorhina rudolphi* | Ribeirão Guaxinduva/Jundiaí/SP/Rio Tietê Basin |
| 95 | LBP 7383 | 34843 | JN689310/ FJ965496/FJ965507/FJ965517 | *Neoplecostomus selenae* | Ribeirão das Batéias/Riacho Grande/SP/Rio Paranapanema Basin |
| 96 | LBP 7525 | 34832 | -/JN089843/JN089818/JN089794 | *Neoplecostomus botucatu* | Cachoeira Véu da Noiva/Botucatu/SP/Rio Paranapanema Basin |
| 97 | NUP 3560 | 9701 | JN689293/ FJ434506/FJ434529/FJ965516 | *Neoplecostomus yapo* | Rio Tibagi/Fortaleza/PR/Rio Paranapanema Basin |
| 98 | LBP 2732 | 17444 | JN689303/FJ965495/FJ965505/FJ965515 | *Neoplecostomus paranensis* | Córrego Mocoquinha/Cajuru/SP/Rio Grande Basin |
| 99 | LBP 5901 | 27990 | JN689304/JN089837/JN089812/JN089788 | *Neoplecostomus langeanii* | Córrego São Domingos/Muzambinho/MG/Rio Grande Basin |
| 100 | LBP 2861 | 18616 | JN689298/JN089833/JN089808/JN089784 | *Neoplecostomus bandeirante* | Rio Paraitinga/Salesópolis/SP/Rio Tietê Basin |
| 101 | NUP 2528 | 9423 | JN689295/FJ434520/FJ434543/FJ965513 | *Neoplecostomus corumba* | Rio Corumbá/GO/Rio Paranaíba Basin/Upper Rio Paraná Basin |
| 102 | LBP 645 | 7593 | JN689311/FJ434508/FJ434531/FJ965518 | *Neoplecostomus microps* | Ribeirão Cajarana/Pindamonhangaba/SP/Rio Paraíba do Sul Basin |
| 103 | LBP 6537 | 31681 | JN689313/FJ965494/FJ965504/FJ965519 | *Neoplecostomus franciscoensis* | Rio das Velhas/Brumadinho/MG/Rio São Francisco Basin |
| 104 | LBP 2551 | 15243 | JN689312/FJ434507/FJ434530/ FJ965512 | *Neoplecostomus espiritosantensis* | Rio Jucu/Domingos Martins/ES/Oriental Coastal Basin |
| 105 | LBP 8380 | 37559 | JN689288/JN089826/JN689277/JN089777 | *Pareiorhina hyptiorhachis* | Rio Pomba/Santa Barbara do Tugúrio/MG/Rio Paraíba do Sul Basin |
| 106 | LBP 1087 | 10256 | JN689290/JN089828/JN089803/JN089779 | *Pareiorhina carrancas* | Córrego do Sapateiro/Barbacena/MG/Rio Grande Basin |
| 107 | LBP 8368 | 37559 | JN689291/JN089829/JN089804/JN089780 | *Pareiorhina carrancas* | Córrego Beijinho/Carrancas/MG/Rio Grande Basin |
| 108 | LBP7385 | 34852 | JN689324/FJ965491/FJ965502/FJ965526 | *Isbrueckerichthys epakmos* | Rio Água Doce/Tapiraí/SP/Rio Ribeira do Iguape Basin |
| 109 | LBP 6389 | 29765 | JN689326/FJ965489/FJ965503/FJ965529 | *Isbrueckerichthys* cf. *calvus* | Rio Taquará/California/PR/Rio Paranapanema Basin |
| 110 | LBP 7373 | 34853 | JN689297/FJ965490/FJ965506/FJ965525 | *Isbrueckerichthys alipionis* | Rio Betari/Iporanga/SP/Rio Ribeira do Iguape Basin |
| 111 | LBP 2650 | 17402 | JN689325/FJ625812/FJ625821/FJ965528 | *Isbrueckerichthys duseni* | Rio Pulador/Campinhos/PR/Rio Ribeira de Iguape Basin |
| 112 | LBP 7384 | 34837 | JN689323/JN089845/JN089820/JN089796 | *Neoplecostomus ribeirensis* | Rio Água Doce/Tapiraí/SP/Rio Ribeira do Iguape Basin |
| 113 | LBP 515 | 6334 | JN689316/FJ965492/FJ965500/FJ965523 | *Kronichthys subteres* | Rio Betari/Iporanga/SP/Rio Ribeira do Iguape Basin |
| 114 | LBP 795 | 8304 | JN689315/FJ434503/FJ434526/FJ965522 | *Kronichthys lacerta* | Rio Marumbi/Morretes/PR/Oriental Coastal Basin |
| 115 | LBP 2122 | 15096 | JN689314/FJ434502/FJ434525/FJ965520 | *Kronichthys heylandi* | Rio Parati-Mirim/Parati/RJ/Oriental Coastal Basin |
| 116 | LBP 1766 | 12886 | JN689317/FJ965493/FJ965501/FJ965521 | *Kronichthys* sp. 1 | Rio Sítio do Meio/Mongaguá/SP/Oriental Coastal Basin |
| 117 | LBP 748 | 8257 | JN689318/FJ625811/FJ625820/FJ965524 | *Pareiorhaphis splendens* | Rio São João/Guaruva/PR/Oriental Coastal Basin |
| 118 | MCP 41275 |  | -/-/EU359438/- | *Pareiorhaphis cameroni* | Rio Cubatão/Águas Mornas/SC/Brazil |
| 119 | LBP 902 | 7989 | JN689319/FJ434514/FJ434537/FJ965532 | *Pareiorhaphis steindachneri* | Rio Itapucu/Jaraguá do Sul/SC/Oriental Coastal Basin |
| 120 | MCP 41909 |  | -/-/EU359437/- | *Pareiorhaphis azygolechis* | Rio Araraquara/Guaratuba/PR/Brazil |
| 121 | LBP 1161 | 8935 | JN689320/FJ434512/FJ434535/FJ965530 | *Pareiorhaphis vestigipinnis* | Rio Caveiras/Painel/SC/Rio Uruguai Basin |
| 122 | LBP 701 | 7363 | JN689321/FJ434513/FJ434536/FJ965531 | *Pareiorhaphis hystrix* | Rio Tainhas/Tainhas/RS/Oriental Coastal Basin |
| 123 | MCP 41747 |  | EU359443/-/-/- | *Pareiorhaphis parmula* | Rio dos Patos/Lapa/PR/Brazil |
| 124 | MCP 41458 |  | EU359439/-/-/- | *Pareiorhaphis eurycephalus* | Rio Rufino/Rio Rufino/SC/Brazil |
| 125 | LBP 4042 | 22905 | KM205167/KM104409/KM104493/- | *Hypoptopoma inexspectatum* | Rio Moa/Cruzeiro do Sul/AC/Brazil |
| 126 | LBP 693 | 7084 | KM205168/KM104410/KM104494/KM104568 | *Hypoptopoma inexspectatum* | Afluente rio Pirai/ Poconé/MT/Brazil |
| 127 | MHNG 2678.015 | PR 12 | JN689282/FJ965486/FJ965498/FJ965508 | *Hypoptopoma inexspectatum* | Rio Paraná/Santa Fé/Argentina |
| 128 | LBP 3081 | 19713 | EU817541/KM104411/KM104495/- | *Hypoptopoma gulare* | Rio Orinoco/Caicara del Orinoco/Bolivar/Venezuela |
| 129 | MHNG 2709.024 | MUS 388 | EU817554/KM104412/KM104496/- | *Hypoptopoma* sp. 1 | Export Iquitos, Upper Amazon basin, Peru |
| 130 | LBP 3165 | 19315 | EU817543/KM104413/KM104497/KM104569 | *Acestridium discus* | Rio Preto da Eva/Rio Preto da Eva/AM/Brazil |
| 131 | LBP 7204 | 35332 | KM205169/KM104414/KM104498/- | *Acestridium* sp. 1 | Igarapé Ya-Mirim/São Gabriel da Cachoeira/AM/Brazil |
| 132 | LBP 6973 | 35324 | KM205170/KM104415/KM104499/KM104570 | *Oxyropsis* sp. 1 | Igarapé Demuriari/São Gabriel da Cachoeira/AM/Brazil |
| 133 | LBP 4300 | 23945 | EU817542/KM104416/KM104500/KM104571 | *Oxyropsis acutirostra* | Igarapé Zamula/Barcelos/AM/Brazil |
| 134 | LBP 2652 | 17407 | EU817545/KM104417/KM104501/- | *Lampiella gibbosa* | Rio Carombé/Campinhos/PR/Brazil |
| 135 | LBP 877 | 8564 | EU817546/KM104418/KM104502/KM104572 | *Otocinclus flexilis* | -/Santo Antônio da Patrulha/RS/Brazil |
| 136 | MCP 25234 |  | -/-/EU370983/- | *Otocinclus arnoldi* | Rio Inhacunda/São Francisco de Assis/RS/Brazil |
| 137 | LBP 5310 | 26831 | KM205171/KM104419/KM104503/- | *Otocinclus hoppei* | Igarapé Uiratapuru/Laranjal do Jari/AP/Brazil |
| 138 | MHNG 2613.057 | CA25 | EU817556/KM104420/KM104504/- | *Otocinclus hoppei* | Arroio Huangana, Alto Pisqui/Loreto/Ucay/Peru |
| 139 | LBP 5132 | 26233 | EU817544/KM104421/KM104505/KM104573 | *Otocinclus vittatus* | Lagoa Bairro Caiçara/Cáceres/MT/Brazil |
| 140 | MCP 34842 |  | -/-/EU359432/- | *Otocinclus cocama* | Afluente do Cano da Concha Supay/Jenaro Herrera/Loreto/Peru |
| 141 | MHNG no number | SU07-350 | EU817558/KM104422/KM104506/- | *Otocinclus mariae* | Witoto Ecu creek/Sipaliwini/Suriname |
| 142 | MHNG 2601.060 | BR98-040 | EU817557/KM104423/KM104507/KM104574 | *Otocinclus mariae* | Rio Acará/-/PA/Brazil |
| 143 | LBP 3510 | 21309 | JN689283/FJ625809/FJ625818/FJ965533 | *Hypostomus nigromaculatus* | Córrego Hortelã/Botucatu/SP/Rio Paranapanema Basin |
| 144 | NUP 1725 | 16652 | KM205172/KM104424/KM104508/- | *Hypostomus microstomus* | Rio Paraná/Guaíra/PR/Brazil |
| 145 | LBP 2544 | 10887 | KM205173/KM104425/KM104509/- | *Hypostomus ancistroides* | Rio Corumbataí/Corumbataí/SP/Brazil |
| 146 | LBP 1557 | 11505 | KM205174/KM104426/KM104510/- | *Rineloricaria lanceolata* | Ribeirão Ínsula/Barra do Garça/MT/Brazil |
| 147 | LBP 5049 | 11506 | KM205175/KM104427/KM104511/- | *Spatuloricaria* sp. 1 | Ribeirão Ínsula/Barra do Garça/MT/Brazil |
| 148 | MCP 31467 |  | -/AY307290/-/- | *Delturus parahybae* | -/Laranjal/MG/Brazil |
| 149 | LBP 2368 | 15363 | JN689278/FJ434499/FJ434524/FJ965535 | *Hemipsilichthys gobio* | Rio Macaquinho/Bairro dos Macacos/SP/Rio Paraíba do Sul Basin |
| 150 | LBP 4956 | 10241 | JN689279/FJ625808/FJ625817/FJ965534 | *Hemipsilichthys papillatus* | Ribeirão da Jacutinga/Bom Jardim de Minas/MG/Rio Paraíba do Sul Basin |
| 151 | LBP 1352 | 11454 | -/KM104428/KM104512/- | *Astroblepus* sp. 1 | Rio Jequetepeque/Magdalena/Cajamarca/Peru |
| 152 | LBP 3284 | 20010 | KM205176/KM104429/KM104513/- | *Astroblepus* sp. 2 | Rio Chorobamba/Huancabamba/Pasco/Peru |
| 153 | LBP 485 | 6040 | -/KM104430/KM104514/- | *Callichthys callichthys* | Córrego do Pombo/Marília/SP/Brazil |
| 154 | LBP 210 | 4134 | -/GU210868/-/- | *Hoplosternum littorale* | Igarapé São Francisco/Rio Branco/AC/Brazil |
| 155 | LBP 2809 | 18894 | GU210997/-/-/- | *Corydoras oiapoquensis* | Guyana coastal rivers/-/-/Guyana |
| 156 | LBP 6862 | 32502 | -/GU210613/-/- | *Corydoras imitator* | Igarapé Puranga/São Gabriel da Cachoeira/AM/Brazil |
| 157 | LBP 449 | 5815 | -/KM104431/EU179801/- | *Diplomystes mesembrinus* | Rio Chubut/Los Altares/Chubut/Argentina |
