# Supplementary material for: Description of a new catfish genus (Siluriformes, Loricariidae) from the Tocantins River basin in central Brazil, with comments on the historical zoogeography of the new taxon
Source: Zookeys. 2016 Jun 14;(598):129–57. doi: 10.3897/zookeys.598.7400 (PMC4926676; doi:10.3897/zookeys.598.7400)
Supplement: Supplementary material 2 — Table S2 [file zookeys-598-129-s002.doc]

**Supplementary table 2.** Models tested to estimate distribution ranges inherited by the descending lineages at each node of the tree. The differences between the models are in the rate of dispersal among adjacent and no adjacent areas. * Represents the model used in the analysis.

|  | **Prediction** | **Dispersal rates between adjacent areas** | **Dispersal rates between no adjacent areas** | **Likelihood** | **AIC** |
| --- | --- | --- | --- | --- | --- |
| M0 – DEC | Dispersal between no adjacent areas not permitted | 1.0 | − | lnL = -179.08 | 362.16 |
| M0 – DEC+J | Dispersal between no adjacent areas not permitted | 1.0 | − | lnL = -166.08 | 338.16 |
| M1 – DEC | Dispersal between no adjacent areas permitted | 1.0 | 1.0 | lnL = -180.43 | 364.87 |
| M1 – DEC + J | Dispersal between no adjacent areas permitted | 1.0 | 1.0 | lnL = -163.85 | 333.70 |
| M2 – DEC | Dispersal between no adjacent areas permitted | 1.0 | 0.5 | lnL = -178.81 | 361.63 |
| *M2 – DEC + J | Dispersal between no adjacent areas permitted | 1.0 | 0.5 | lnL =-162.36 | 330.72 |
| M3 – DEC | Dispersal between no adjacent areas permitted | 0.5 | 0.0001 | lnL = -179.07 | 362.15 |
| M3 – DEC+J | Dispersal between no adjacent areas permitted | 0.5 | 0.0001 | lnL = -166.05 | 338.11 |
| M4 – DEC | Dispersal between no adjacent areas permitted | 0.1 | 0.0001 | lnL = -179.07 | 362.14 |
| M4 – DEC+J | Dispersal between no adjacent areas permitted | 0.1 | 0.0001 | lnL = -165.96 | 337.93 |
| M5 – DEC | Dispersal between no adjacent areas permitted | 0.01 | 0.0001 | lnL = -178.98 | 361.96 |
| M5 – DEC+J | Dispersal between no adjacent areas permitted | 0.01 | 0.0001 | lnL = -165.20 | 336.41 |
